# Coenzyme Q_0_ inhibited the NLRP3 inflammasome, metastasis/EMT, and Warburg effect by suppressing hypoxia-induced HIF-1α expression in HNSCC cells

**DOI:** 10.7150/ijbs.93943

**Published:** 2024-05-05

**Authors:** Hsin-Ling Yang, Che-Wei Chang, Chithravel Vadivalagan, Sudhir Pandey, Siang-Jyun Chen, Chuan-Chen Lee, Jhih-Hsuan Hseu, You-Cheng Hseu

**Affiliations:** 1 Institute of Nutrition, College of Health Care, China Medical University, Taichung 406040, Taiwan.; 2 Department of Surgery, University of Michigan Medical Center, Ann Arbor, Michigan 48109, United States.; 3 Department of Cosmeceutics, College of Pharmacy, China Medical University, Taichung 406040, Taiwan.; 4 Department of Health and Nutrition Biotechnology, Asia University, Taichung 413305, Taiwan.; 5 Department of Dermatology, Chang Gung Memorial Hospital, Kaohsiung 83301, Taiwan.; 6 Chinese Medicine Research Center, China Medical University, Taichung 404333, Taiwan.; 7 Research Center of Chinese Herbal Medicine, China Medical University, Taichung 404333, Taiwan.

**Keywords:** CoQ_0_, HIF-1α, NLRP3, EMT, Warburg effect

## Abstract

Coenzyme Q_0_ (CoQ_0_), a quinone derivative from *Antrodia camphorata*, has antitumor capabilities. This study investigated the antitumor effect of noncytotoxic CoQ_0_, which included NLRP3 inflammasome inhibition, anti-EMT/metastasis, and metabolic reprogramming via HIF-1α inhibition, in HNSCC cells under normoxia and hypoxia. CoQ_0_ suppressed hypoxia-induced ROS-mediated HIF-1α expression in OECM-1 and SAS cells. Under normoxia and hypoxia, the inflammatory NLRP3, ASC**/**caspase-1, NFκB, and IL-1β expression was reduced by CoQ_0_. CoQ_0_ reduced migration/invasion by enhancing epithelial marker E-cadherin and suppressing mesenchymal markers Twist, N-cadherin, Snail, and MMP-9, and MMP-2 expression. CoQ_0_ inhibited glucose uptake, lactate accumulation, GLUT1 levels, and HIF-1α-target gene (HK-2, PFK-1, and LDH-A) expressions that are involved in aerobic glycolysis. Notably, CoQ_0_ reduced ECAR as well as glycolysis, glycolytic capability, and glycolytic reserve and enhanced OCR, basal respiration, ATP generation, maximal respiration, and spare capacity in OECM-1 cells. Metabolomic analysis using LC-ESI-MS showed that CoQ_0_ treatment decreased the levels of glycolytic intermediates, including lactate, 2/3-phosphoglycerate, fructose 1,6-bisphosphate, and phosphoenolpyruvate, and increased the levels of TCA cycle metabolites, including citrate, isocitrate, and succinate. HIF-1α silencing reversed CoQ_0_-mediated anti-metastasis (N-Cadherin, Snail, and MMP-9) and metabolic reprogramming (GLUT1, HK-2, and PKM-2) under hypoxia. CoQ_0_ prevents cancer stem-like characteristics (upregulated CD24 expression and downregulated CD44, ALDH1, and OCT4) under normoxia and/or hypoxia. Further, in IL-6-treated SG cells, CoQ_0_ attenuated fibrosis by inhibiting TGF-β and Collagen I expression and suppressed EMT by downregulating Slug and upregulating E-cadherin expression. Interesting, CoQ_0_ inhibited the growth of OECM-1 tumors in xenografted mice. Our results advocate CoQ_0_ for the therapeutic application against HNSCC.

## Introduction

Over 800,000 people worldwide have head and neck squamous cell carcinoma (HNSCC). Papillomavirus infection and tobacco and alcohol consumption are the primary etiological agents that are associated with the pathogenesis of HNSCC 1. The transcriptional response is epigenetically regulated by low oxygen levels under hypoxic conditions, leading to a poor prognosis in cancer. Interestingly, HIF-1α-regulated genes are expressed under both normoxic and hypoxic conditions; therefore, they could be targeted to ameliorate solid cancers 2. HNSCC cells exposed to hypoxia are much more resistant to radiotherapy than their normoxic counterparts 3. Therefore, developing molecular therapies that target cancer hypoxia is an amenable treatment approach for HNSCC patients.

Higher NOD-like receptor (NLR) family pyrin domain-containing 3 (NLRP3) inflammasome levels in cancer cells are linked with increased HIF-1α expression 4. Expression of Nuclear factor κB (NFκB), Interleukin 1β (IL-1β), and caspase-1 is necessary for NLRP3 inflammasome activation 5. Tumor etiology involves dysregulated activation of the NLRP3 inflammasome, although its significance in the initiation and development of cancers is still controversial given the contradictory results thus far. A few studies have suggested that blocking the NLRP3 inflammasome/IL-1β pathway may be a potential strategy for treating HNSCC by ameliorating the tumor microenvironment 6.

Increased expression of HIF-1α in HNSCC cells controls the expression of genes related to epithelial-mesenchymal transition (EMT). The EMT in cancer cells is a dynamic biological process that is involved in migration/invasion and metastasis. EMT has been related to the metastasis potential of tumors and gives cancer cells the capacity to migrate to other body parts by increasing their invasive ability. Extracellular matrix components, cytoskeletal proteins, transcription factors, and cell-surface proteins are a few of the protein types that affect EMT 7. The clinical significance of EMT prediction in HNSCC cells has been highlighted, indicating its value in treating head and neck cancer. An integrative EMT signature analysis was used to establish an EMT prediction model that is unique to HNSCC 8.

HIF-1α is a key factor that is responsible for regulating the energy supply in tumor cells under both normoxic and hypoxic conditions 9. Tumor cells require enhanced ATP delivery, protein synthesis, lipid synthesis, and DNA duplication for increasing prevalence, and these effects are achieved through the Warburg effect, which encourages aerobic glycolysis over tricarboxylic acid cycle (TCA) cycle oxidative phosphorylation. The Warburg effect describes how cancer cells absorb glucose and produce more lactic acid than normal cells when there is plenty of oxygen present. This highlights the metabolic alterations that cancer cells acquire in order to produce more ATP and metabolic intermediates, which power the synthesis of biological macromolecules and aid in cell survival, development, and multiplication. An acidic microenvironment is created by pyruvate, an end product of glycolysis that preferentially accumulates in cancer cells and is transformed to lactic acid by lactate dehydrogenase, which promotes the growth and invasion of cancer cells 10, 11. In HNSCC, there is an adverse correlation between high lactic acid levels and overall patient survival 12. The aerobic glycolysis is notably enhanced, which is associated with tumor evolution and reduced susceptibility to chemotherapy in HNSCC. Solid cancers, including HNSCC, undergo metabolic reprogramming to support enhanced growth requirements.

The indigenous Taiwanese medicinal mushroom *Antrodia camphorata* is used as traditional medicine in the treatment of numerous illnesses, such as a number of malignancies, common skin ailments, indigestion, liver dysfunction, hypertension, and enteric diseases 13. Numerous characteristics, including antioxidant, anti-angiogenesis, anti-inflammation, and anti-tumorigenesis, were attributed to the submerged fermented broths (mycelium and fruiting body) of *Antrodia camphorata* 13. In our prior work, we identified that fermented broths of *Antrodia camphorata* prepared from submerged cultures contained high concentrations of Coenzyme Q_0_ (CoQ_0_, 17.3%) detected by a high-performance liquid chromatography (HPLC) analysis 14. CoQ_0_ (ubiquinone 0; 2,3 dimethoxy-5-methyl-1,4 benzoquinone) is a ubiquinone chemical that is redox-sensitive as well as a constituent of the mitochondrial respiratory chain 15, 16. In our previous investigation, results revealed the anti-inflammatory and antiangiogenic properties of CoQ_0_ both *in vivo* and *in vitro* 17.

Coenzyme Q_0_ (CoQ_0_), which is a quinone derivative from *Antrodia camphorata*, has therapeutic effects against various cancer cells, such as A549, HepG2, SW480, B16F10, MDA-MB-231, and SKOV-3 cells 18-20. Our previous research demonstrated that CoQ_0_ prevents EMT/metastasis, apoptosis, and/or autophagy in glioblastoma cells, TWIST1-overexpressing HNSCC cells, triple-negative breast cancer cells, and ovarian carcinoma cells 20-22. This investigation studied the molecular mechanisms and therapeutic effects of *Antrodia camphorata*-derived CoQ_0_ on the NLRP3 inflammasome, EMT/metastasis, and Warburg effect (metabolic reprogramming) in HNSCC (OECM-1 and SAS) cells.

## Materials and Methods

### Reagents and antibodies

GIBCO (BRL/Invitrogen, Carlsbad, USA) supplied the antibiotics (penicillin/streptomycin/neomycin) and DMEM. CoQ_0_ (2,3-dimethoxy-5-methyl-1,4-benzoquinone, purity > 99%) was obtained from Sigma-Aldrich (St. Louis, MO, USA). Antibodies against the following targets were obtained: IL-1β (ab9722) (Biorbyt, and Abcam, Cambridge, UK), NFκB (GTX 107678), (GeneTex, Irvine, USA), NLRP3 (#15101), Vimentin (#5741), HIF-1α (#79233), N-cadherin (#13116), Apoptosis-associated speck-like protein (ASC) (#13833), Snail (#3879), Matrix metalloproteinase-2 (MMP-2) (#4022), Matrix metalloproteinase-9 (MMP-9) (#3852), Octamer-binding transcription factor 4 (OCT4) (#4286), Aldehyde dehydrogenase 1 (ALDH1) (#12035), CD44 (#3570), Slug (#9585), pyruvate kinase M-2 (PKM-2) (#4053), and Twist (#69366) (Cell Signaling Technology, Danvers, USA) Caspase-1 (sc-56036), Glucose transporter 1 (GLUT1) (sc-377228), Hexokinase 2 (HK-2) (sc-374091), Lactate dehydrogenase A (LDH-A) (sc-137246), Phosphofructokinase 1 (PFK-1) (sc-293072), TGF-β (sc-130348), CD24 (sc-19585), E-cadherin (sc-8426), glyceraldehyde-3-phosphate dehydrogenase (GAPDH) (sc-47724) and β-actin (sc-47778) (Santa Cruz Biotechnology, Heidelberg, Germany). 3-[4,5-dimethylthiazol-2-yl]-2,5 diphenyl tetrazolium bromide (MTT) (Sigma-Aldrich, St. Louis, MO, USA), Diamidino-2-phenylindole dihydrochloride (DAPI, Calbiochem-La Jolla, USA), 2-deoxy-2-[(7-nitro-2,1,3-benzoxadiazol-4-yl) amino]-D-glucose (2-NBDG), and the L-lactate assay kit (Cayman Chemical Company, MI, USA) were used, and the extracellular acidification rate (ECAR) and oxygen consumption rate (OCR) were examined through a Seahorse XF assay (Agilent Technologies, USA). Cobalt chloride** (**CoCl_2_) and other reagents were obtained from Sigma-Aldrich (St. Louis, MO, USA).

### Preparation of the fermented culture broths of *Antrodia camphorata*

*Antrodia camphorata* was collected from Nantou County, Taiwan. All AC specimens used were saved in the CMU repository and named “CMU-AC010”. Dr. Shy-Yuan Hwang from 'The Endemic Species Research Institute' in Nantou, Taiwan, characterized the fermented broths prepared from *Antrodia camphorata* 14. The original colonies acquired above were plated on agar potato dextrose media at 30°C and incubated for 15-20 days to establish the seed cultures of *Antrodia camphorata*. Next, the whole colony was added into a 50-mL of sterile water flask and homogenized, following which the mycelial suspension was used as an inoculum for subsequent culture broth preparation. The initiating culture was then generated in a 20-liter fermenter (BioTop) that was whirled at 150 rpm and maintained at 30°C with a 0.2 vvm aeration rate. A 250-liter agitated fermenter (BioTop) was inoculated with 15 L of mycelium inoculum from a 5-day culture, and the mixture was then incubated for 331 h with an aeration rate of 0.075 vvm. The mycelia and deep-red fermented culture broths were separated by passing them through a 20-mesh sieve. After being centrifuged for 10 min at 3000 g, the culture soup was filtered through a 0.2 µm pore-size filter. Three to four separate sets of the fermented *Antrodia camphorata* cultures were utilized for experimental purposes. Then, the obtained filtrate was concentrated and lyophilized into a powder. A dry matter yield of 9.72 g/L was obtained from the culture broths. The aqueous solution was prepared by dissolving the powder samples at 25°C in 10 mM sodium phosphate buffer (pH 7.4) containing 0.15 M sodium chloride. For further investigation, the stock solutions were prepared and kept at -20°C.

### CoQ_0_ isolation from the fermented broths of *Antrodia camphorata* by HPLC analysis

The fermented broths of *Antrodia camphorata* from the submerged cultures were subjected to HPLC characterization using a 4.6 x 250 mm RP-18 column (COSMOSIL, 5C_18_-AR-II, Waters) at a flow rate of 1.0 mL/min and detection at UV 254 and 220 nm. A standard solution for HPLC analysis was prepared by dissolving the *Antrodia camphorata* fermented culture broths in water (5.0 mg/mL) and filtering it through a 0.22 µm membrane filter. A gradient elution of 5-60% was obtained with the mobile phase consisting of (A) 0.05% trifluoroacetic acid (TFA) and (B) acetonitrile applied for 5-40 min. 20 µL aliquots were injected with a flow rate of 1.0 mL/min. The key constituents of the fermented broths of *Antrodia camphorata* were analyzed using chromatography and spectrum analysis. C_5_D_5_N was used as the measuring solvent for NMR 1D and 2D spectra on an NMR spectrometer (Bruker, Unity Plus 400 MHz) using a Shimadzu LC program for all analyses. The system incorporated an autosampler (SIL-20), a PDA detector (SPD-M20A), a pump (LC-20AT), and a column oven (CTO-20A). A mass Esquire HCT (Bruker), an Agilent 1100 series, and a column (Agilent, Zorbax Eclipse, SB-C18, 2.1 x 150 mm, 5 µm) were used for the LC-MS analyses. Multiple chromatographic peaks were obtained, indicating the presence of a diverse range of chemicals in the fermented broths of *Antrodia camphorata*. A yield of 17.3% (at 254 nm) and 13.5% (at 220 nm), respectively, was obtained for CoQ_0_ in the fermented culture broths of *Antrodia camphorata* 14.

### Cell culture

HNSCC OECM-1, SAS, Cal-27, HSC-3, and FaDu cells and human salivary gingival epithelial SG cells were obtained from the American Type Culture Collection (VA, USA). OECM-1 cells were cultured in RPMI-1640, FaDu and Cal-27 cells were cultured in DMEM, and SAS and HSC-3 cells were cultured in DMEM/F12, and heat-inactivated fetal bovine serum (FBS, 10%) was supplemented into these media, along with penicillin-streptomycin-neomycin (1%), and glutamine (2 mM). The cells were maintained in a 5% CO_2_ and 37 °C humidified incubator under normoxia or hypoxia (1% O_2_). Interleukin 6 (IL-6)-treated SG cells were grown in DMEM supplemented with heat-inactivated FBS (1%), 1% penicillin-streptomycin-neomycin (1%), and glutamine (2 mM), and they were maintained in a humidified incubator with 5% CO2 at 37 °C.

### MTT assay

Cell viability was spectrophotometrically measured using MTT assays as described earlier with minor modifications 23. 4 × 10^4^ cells/well (20-40 passages) were cultivated in 24-well plates and treated with the indicated concentration of CoQ0 for 24, 48, or 72 h. The MTT reagent (400 μL 0.5 mg/mL MTT in PBS) was added to each well and incubated for 2 h at 37 °C, following which the formed MTT formazan crystals were solubilized using an equal volume of DMSO (400 μL). Finally, an ELISA microplate reader (Bio-Tek Instruments Inc., Winooski, USA) was employed to read the absorbance (A) at 570 nm. The assay was executed in triplicate at each concentration. The cell viability was expressed as the proportion of viable cells relative to vehicle-treated cells (A_570_ of treated cells/A_570_ of untreated cells) × 100.

### Colony formation assay

To examine the colony formation ability of OECM-1 and SAS cells, a colony formation assay using previously established procedures was performed 22. In brief, OECM-1 and SAS cells (3 × 10^4^ cells/well of a 6-well plate) were exposed to CoQ_0_ at concentrations of 0-7.5 μM for 24 h. All the assays were performed in triplicate, and the colony formation ability was expressed as a percentage relative to the number of colonies that were formed in the absence of CoQ_0_ (as 100%). A bright field microscope (100X) was used to visualize the colonies and acquire the images.

### Immunoblotting assay

Cells were seeded at a density of 1 × 10^6^ cells/10-cm dish (20-40 passages), and the next day, they were treated with CoQ_0_ (0-7.5 μM) for 24 h. After treatment, the cells were rinsed with ice-cold phosphate buffer saline (PBS), and nuclear, cytoplasmic, and total proteins were extracted according to the manufacturer's recommendations (Pierce Biotechnology, Rockford, IL, USA). The specified proteins were analyzed using established immunoblotting procedures, as previously described 22. Bovine serum albumin was used as the standard to estimate the protein content of each sample using the Bio-Rad protein assay reagent. Equal portions of denatured protein samples (50-100 µg) were electrophoresed on an 8-15% SDS-PAGE gel and then transferred overnight to a polyvinylidene difluoride (PVDF) membrane. 5% nonfat dry milk powder in Tris-buffered saline (0.1% Tween 20, TBST) was used to block the non-specific binding of proteins on the membrane for 30 minutes at room temperature. Following incubation at 4°C with primary antibodies overnight, the membranes were exposed to an HRP-conjugated goat anti-mouse or anti-rabbit secondary antibody for 2 h. The protein bands were detected using the enhanced chemiluminescence substrate (Bio-Rad, Hercules, CA, USA). The protein spot intensities were analyzed using commercially accessible software (AlphaEase, Genetic Technology Inc., Miami, FL, USA). Later, the PVDF membrane was de-probed of prior primary antibodies using the membrane stripping procedure and re-probed for specific proteins. The following chemicals made up the stripping buffer: glycine (1.5%), SDS (0.1%), Tween (20%), and pH (2.2). A suitable volume of the stripping buffer was applied to cover the membrane surface, and it was gently rocked for 15 minutes at room temperature. The step was repeated three times, following a fresh addition of stripping buffer, and then the buffer was discarded. The membrane was then rinsed three times (each for 10 minutes) using TBST. Next, the membrane was blocked with 5% nonfat dry milk powder as above at room temperature for 30 minutes, followed by incubation with primary antibodies.

### Measurement of ROS production using DCFH2-DA assay

The production of intracellular oxygen radicals was measured using DCFH_2_-DA, as previously described 24. In a 12-well plate, 2 × 10^5^ cells/well were treated with CoQ_0_ (7.5 µM) for 0-90 min. Afterward, the cells were rinsed with PBS and incubated with 10 µM DCFH_2_-DA for another 30 min at 37 °C in serum-free culture medium. Next, PBS was used to wash the cells, and the fluorescence intensity of DCF indicating ROS levels was analyzed using fluorescence microscopy (Olympus, Center Valley, PA, USA).

### Annexin V-FITC and PI staining for apoptosis analysis

The apoptosis of OECM-1 and SAS cells (1x10^6^ cells/10-cm dish) (20-40 passages) was examined using Annexin V-FITC (BD Biosciences, MI, USA) and PI (propidium iodide) (Sigma-Aldrich, St. Louis, MO, USA) staining as per manufacturer instructions. Summarily, cells were incubated with CoQ0 (0-7.5 μM) for 24 h, trypsinized and washed twice with PBS, followed by centrifugation at 800 rpm for 5 min. Then, the resulting fluorescence intensity [green (FITC) and red (PI)] of each sample was quantified using a FACSCalibur flow cytometer (Accuri C6, BD Biosciences, MI, USA) and Cell Quest software. The results attained were construed as follows: (Q1) PI-positive, Annexin V-FITC-negative stained cells were adjudicated to be in necrosis; (Q2) PI-positive, Annexin V-FITC-positive stained cells were adjudicated to be in late apoptosis; (Q3) cells negative for both PI and Annexin V-FITC staining were accepted as normal live cells; and (Q4) PI-negative, Annexin V-FITC-positive stained cells were arbitrated to be in early apoptosis.

### Detection of CD24 and TGF-β expression by flow cytometry

In a 10-cm dish, cells (1 × 10^6^ cells/dish) were grown and exposed to CoQ_0_ (0-7.5 μM) for 24 h. Afterwards, the cells were washed and trypsinized, and centrifugation for 5 minutes at 1500 rpm was used to collect the cell pellet. Next, the cells were subjected to fixation and permeabilization using the Cytofix/Cytoperm Kit (BD Biosciences, MI, USA) and rinsed with PBS. Next, the cells were incubated for 1 h at room temperature with primary human monoclonal anti-TGF-β and anti-CD24 antibodies that were conjugated to PE (BD Biosciences, MI, USA) before being rinsed three times with PBS. PE-positive cells were evaluated by an Accuri C6 Flow Cytometer (BD Biosciences, MI, USA).

### Cell migration assay

A standardized procedure 25 with minor modifications was used to investigate the effect of CoQ_0_ on cell migration using a wound-healing assay. Briefly, cells were seeded in a 1% gelatin-coated 12-well plate with an Ibidi culture insert (2 × 10^4^ cells/well). The cells were pretreated with IL-6 (50 ng/mL) and then exposed to CoQ_0_ (0-7.5 μM for 24 or 48 h) in 1% FBS medium before being washed with PBS. The cells were subjected to staining with Giemsa solution (Merck, Darmstadt, Germany), and a phase-contrast microscope (200 × amplification) was used to assess the migrated cells, which were analyzed using Image-Pro Plus software (Media Cybernetics, Inc., USA).

### Cell invasion assay

Matrigel invasion chambers (BD Biosciences, MI, USA) were used to assess cell invasion following previously standardized procedures 26. For invasion assay, 10 μL of Matrigel (25 mg/50 mL) were coated onto the 8-μm polycarbonate membrane filters, and then the cells (2 × 10^4^ cells/well in 12-well plates) were seeded onto the Matrigel-treated filter in 200 μL of CoQ_0_ (0-7.5 μM) for 24 h deprived of serum. Cell migration or Matrigel invasion underwent 24 h incubation at 37ºC. The cells that did not migrate were detached from atop the membrane by using a cotton swab after 24 h. On the reverse side of the membrane, the cells that migrated were fixed with ice-cold methanol (75%) for 15 min and subsequently, washed with PBS three times. Following the experimental treatments, the cells were subjected to Giemsa staining, and after being rinsed with PBS, the invading cells were imaged using an optical microscope (200 X) and manually quantified.

### Immunofluorescence assay

The cells were seeded (3x10^4^ cells/8-chamber slide) in 1% FBS-supplemented medium overnight and exposed to CoQ_0_ (0-7.5 μM) for 24 h treatment. At the end of the specified treatments, immunofluorescence staining procedures were followed as previously described 24. Post treatments, the cells were fixed using 2% paraformaldehyde for 15 min and then permeabilized for 10 min with 0.1% Triton X-100 and then washed and blocked with 10% FBS in PBS. The cells were then incubated with primary antibodies (anti-HIF-1α, anti-E-cadherin, and anti-HK-2) in 1.5% FBS for 2 h. The secondary antibodies conjugated with fluorophores Alexa Fluor 594 (Red) or Alexa Fluor 488 (Green) (Invitrogen Life Technologies, Gaithersburg, MD) were used for 1 h at room temperature. Lastly, DAPI (1 μg/mL) for 5 min was used to stain the nuclei of cells. Then, the cells were rinsed with PBS, and images were acquired using a fluorescence microscope and quantified using ImageJ analysis software (National Institutes of Health, USA).

### Glucose uptake analysis

Glucose utilization was measured following a previously described protocol 27 with a Glucose Uptake Assay Kit (Cayman, US). The fluorescently tagged glucose derivative 2-NBDG was used to examine the glucose absorption capacity of OECM-1 cells. The cells (1×10^5^ cells/well of a 24-well plate) were grown in RPMI-1640 medium without carbon sources. After being exposed to CoQ_0_ (0-7.5 μM) for 6 h, the cells were gently washed with PBS and then incubated for 30 min at 37 °C with 100 μM 2-NBDG in glucose-free medium. Then, the cells were rinsed with PBS, and images were immediately captured using a fluorescence microscope (Ex_(λ)_/Em_(λ)_: 485/530 nm).

### Estimation of lactate production

Extracellular and intracellular lactate production by OECM-1 cells was estimated using the L-Lactate Assay Kit following previously established procedures 28. At the end of the designated treatments, the lactate concentration within the culture medium (extracellular) and cell pellet (intracellular) samples was determined.

### Determination of extracellular acidification rate (ECAR)

OECM-1 cells (3 × 10^4^ cells/well) were seeded in the Seahorse XFp culture XF24 well overnight, and then the cells were exposed to CoQ_0_ (5 μM) without or with the hypoxia-mimetic agent CoCl_2_ (10 μM) for 24 h. The Seahorse test was performed in an XFp Analyzer System (Agilent Technologies, CA, USA) following the manufacturer's instructions. For consistency of data, treatment with CoQ_0_ (5 μM) alone or CoQ_0_ (5 μM) + CoCl_2_ (10 μM) was performed in the same plate (24-well) of OECM-1 cells. Before tests using the XFp Analyzer, the treated cells were changed to a specific medium (without glucose, FBS, sodium bicarbonate, and HEPES) and incubated in an incubator without CO_2_ (at 37°C) for 30-60 min. To measure glycolytic activity, the following injections were sequentially administered: glucose (10 mM), oligomycin (1.0 µM), and 2-deoxyglucose (2-DG, 50 mM). Measurements were collected after each injection. After detection of the extracellular acidification ratio, the rise in ECAR was correlated with the rate of glycolysis, glycolytic capacity, and glycolytic reserve under baseline conditions.

### Assessment of oxygen consumption rate (OCR)

The OCR was measured using the Seahorse test with an XFp Analyzer System (Agilent Technologies, CA, USA) according to the manufacturer's instructions. In brief, OECM-1 cells (3 × 10^4^ cells/well) were grown in Seahorse XFp culture XF24 wells overnight, followed by exposure to CoQ_0_ (5 μM) with or without CoCl_2_ (10 μM) for 24 h. Next, the cells were cultured for 1 h at 37 °C in a CO_2_-free incubator in Seahorse assay medium. An XFp Analyzer System was used to run the Seahorse test, and the following injections were sequentially administered: oligomycin (1.0 μM), rotenone/antimycin A (0.5 μM), and carbonyl cyanide-p-(trifluoromethoxy) phenylhydrazone (FCCP, 0.5 μM). Readings were collected after each injection.

### Metabolomic analysis

The cells were grown on 6-well plates (2 × 10^5^ cells/well) and treated with CoQ_0_ (2.5-5 µM) as previously described. The samples were prepared, and analysis was performed using liquid chromatography‒electrospray ionization tandem mass spectrometry (LC-ESI-MS) following previously described procedures 21. The measurement of the metabolites in the samples was performed using a negative ion mode LC-ESI-MS platform, and the data were evaluated by UNIFI software (Waters).

### HIF-1α siRNA transfection

OECM-1 and SAS cells (5 × 10^5^ cells/well) were grown in 6-well plates, and transfection was performed when the cells reached 60-70% confluence. The transfection of siRNAs targeting HIF-1α was performed using a commercially available transfection kit (Lipofectamine RNAiMAX, Invitrogen, Carlsbad, CA, USA) according to the manufacturer's recommendations. The culture medium was then substituted with 500 μL of Opti-MEM on the following day and the cells were transfected using RNAiMAX transfection reagent. 5 μL RNAiMAX was mixed with 250 μL of Opti-MEM and incubated the mixture for 5 min at room temperature. In another tube, siRNA (100 pM) was prepared, and mixed with the tube comprising 250 μL of Opti-MEM. The mixture, thus obtained, was poured into the diluted RNAiMAX. The siRNA/RNAiMAX concoction (500 μL) was allowed to incubate for additional 25 min at room temperature to form a transfection complex. Subsequently, the obtained complex mixture was added to the 6-well plate-making the final transfection volume to 1 mL. At 6 h post-transfection, the medium was replaced with 2 mL standard culture medium and grown at 37°C. After siHIF-1α transfection, OECM-1 and SAS cells were exposed to CoQ_0_ (0 or 7.5 μM) for 24 h.

### Animal model and tumor xenograft preparation

Eight-week-old male athymic nude mice (BALB/c-*nu*) were acquired from the National Laboratory Animal Center (Taipei, Taiwan) and housed in discrete cages in a pathogen-free secluded facility with a 12-h light/dark cycle; the mice were given free access to rodent chow and water. This study was approved by the Animal Ethics Research Board of China Medical University (Approval no. IACUC-2018-065). For tumor xenograft preparation, the left rear flanks of BALB/c nude mice were subcutaneously injected with OECM-1 cells (5 × 10^6^ cells in 100 μL of matrix gel). The treatment groups were orally administered 1.5 mg/kg of CoQ_0_ every two days for 28 days, and the control group was injected with vehicle alone (1% DMSO) on the same schedule. Body weight measurements were recorded twice every seven days to track the side effects caused by the drugs. The tumor volume was measured every four days using caliper measurements and with the following formula: length × width^2^ × 0.5 29. The mice were sacrificed on the 28^th^ day of treatment. Any signs of treatment side effects on vital organs were examined by a veterinary pathologist.

### Statistical analysis

The normal distribution of data was determined relative to the deviation from the mean values of group data. For normally distributed data, one-way analysis of variance (ANOVA) with Dunnett's test was used for pair-wise comparisons and to analyze the significant differences with the control group. The results were presented as the mean ± standard deviation (n=3). β-actin, or GAPDH, served as the internal control. *p < 0.05; **p < 0.01; ***p < 0.001 compared with untreated cells. ^#^*p* < 0.05; ^##^*p* < 0.01; ^###^*p* < 0.001 compared with normoxia-, CoCl_2_-, or IL-6-treated cells.

## Results

### CoQ_0_ suppresses cell viability, colony formation, and HIF-1α expression in HNSCC (OECM-1 and SAS) cells

**Figure 1:**
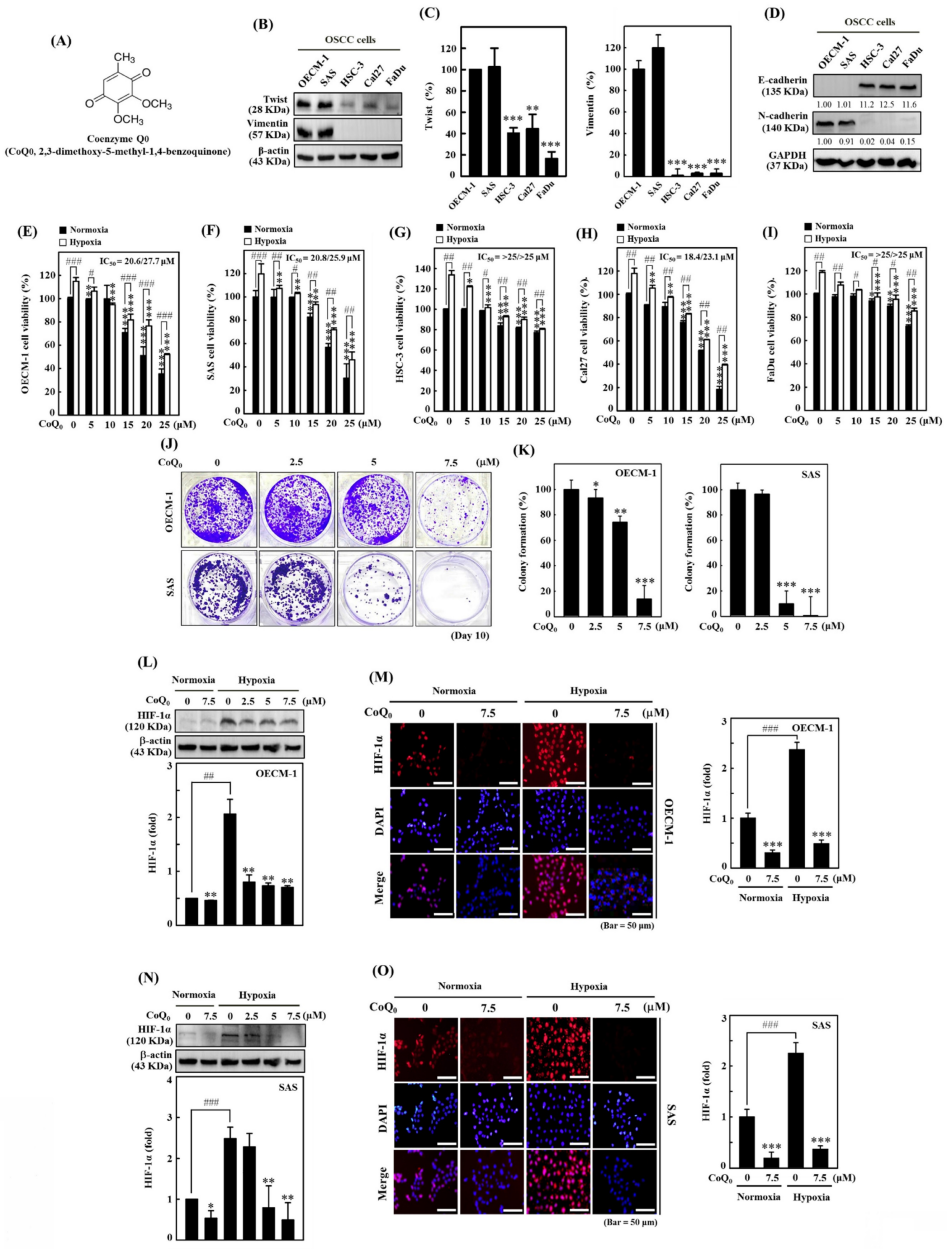
Coenzyme Q_0_ (CoQ_0_) inhibits cell viability, colony formation, and HIF-1α expression in HNSCC cells.** (A)** Chemical structure of CoQ_0_, a quinone derivative from *Antrodia camphorata.* **(B-C)** Immunoblotting showing Twist and Vimentin expression in OECM-1, SAS, Cal-27, HSC-3, or FaDu cells. **p < 0.01; ***p < 0.001 compared with OECM-1 cells. **(D)** N-cadherin and E-cadherin expression in OECM-1, SAS, HSC-3, Cal-27, or FaDu cells as demonstrated by Western blotting. **(E-I)** Cell viability was assessed using an MTT colorimetric assay. OECM-1, SAS, HSC-3, Cal-27, and FaDu cells were treated with CoQ_0_ (0-25 μM for 24 h) under normoxic and hypoxic (1% O_2_) conditions. **(J-K)** OECM-1 and SAS cells were treated with CoQ_0_ (0-7.5 μM) and incubated for 8 days to assess colony formation. **(L-O)** HIF-1α expression was examined by immunoblotting and immunofluorescence analysis. OECM-1 **(L-M)** and SAS **(N-O)** cells were treated with CoQ_0_ (0-7.5 μM for 24 h) under normoxia and hypoxia. Values are the mean ± SD (n=3). *p < 0.05; **p < 0.01; ***p < 0.001 compared with untreated cells. ^#^*p* < 0.05; ^##^*p* < 0.01; ^###^*p* < 0.001 compared with normoxia-treated cells.

Our previous investigation showed that CoQ_0_ (**Figure 1A**), which is a novel quinone derived from* Antrodia camphorata*, exerted *in vitro* and *in vivo* antitumor effects against Twist-overexpressing HNSCC (OECM-1 and FaDu-Twist) cells via the ROS-modulated induction of apoptosis and autophagy 30. EMT is a dynamic biological process that is indispensable for tumor cell metastasis and involves the diminution of epithelial proteins (E-cadherin) and the increment of mesenchymal proteins (Twist, Vimentin, and N-Cadherin). Our findings revealed that Twist and Vimentin expression were higher in HNSCC (OECM-1 and SAS) cells than in other HNSCC (HSC-3, Cal-27, and FaDu) cells (**Figure 1B-C**). Additionally, the expression of the mesenchymal marker N-cadherin was higher, while the expression of the epithelial marker E-cadherin was lower in OECM-1 and SAS cells than in other HNSCC (HSC-3, Cal-27, and FaDu) cells (**Figure 1D**). These results suggested that OECM-1 and SAS cells have more EMT/metastasis potential than HSC-3, Cal-27, and FaDu cells.

The MTT assay showed that CoQ_0_ administration markedly reduced the cell viability of OECM-1, SAS, FaDu, HSC-3, and Cal-27 cells under normoxic and hypoxic conditions, with IC_50_ values of 20.6/27.7, 20.8/25.9, >25/>25, 18.4/23.1, and >25/>25 μM, respectively (**Figure 1E-I**). Notably, cytotoxicity in HNSCC cells was more prominent under normoxia than under hypoxia (**Figure 1E-I**). To examine the prolonged effect of CoQ_0_ (0-7.5 µM for 10 days) on cellular proliferation, the colony forming capacity (a feature of cancer cells that is strongly associated with tumorigenesis) was investigated. CoQ_0_ administration dose-dependently inhibited the capacity of OECM-1 and SAS cells to form colonies (**Figure 1J-K**).

In cancer cells, HIF-1α expression is related to the NLRP3 inflammasome, the Warburg effect, and EMT/metastasis 31. The study investigated the efficacy of CoQ_0_ (0-7.5 µM for 24 h) on HIF-1α expression in OECM-1 and SAS cells under both normoxia and hypoxia (1% O_2_). Based on the results of immunoblotting and immunofluorescence analysis, we found that HIF-1α expression in OECM-1 (**Figure 1L-M**) and SAS (**Figure 1N-O**) cells was higher under hypoxia than normoxia, while CoQ_0_ significantly inhibited HIF-1α expression under both normoxia and hypoxia. Our results validated the use of a noncytotoxic CoQ_0_ dose ranging from 0 to 7.5 µM to inspect the effect of CoQ_0_ on HIF-1α inhibition, NLRP3 inflammation suppression, EMT/metastasis inhibition, and metabolic reprogramming in OECM-1 and SAS cells.

### CoQ_0_ inhibits hypoxia-induced intracellular ROS production in OECM-1 and SAS cells

**Figure 2:**
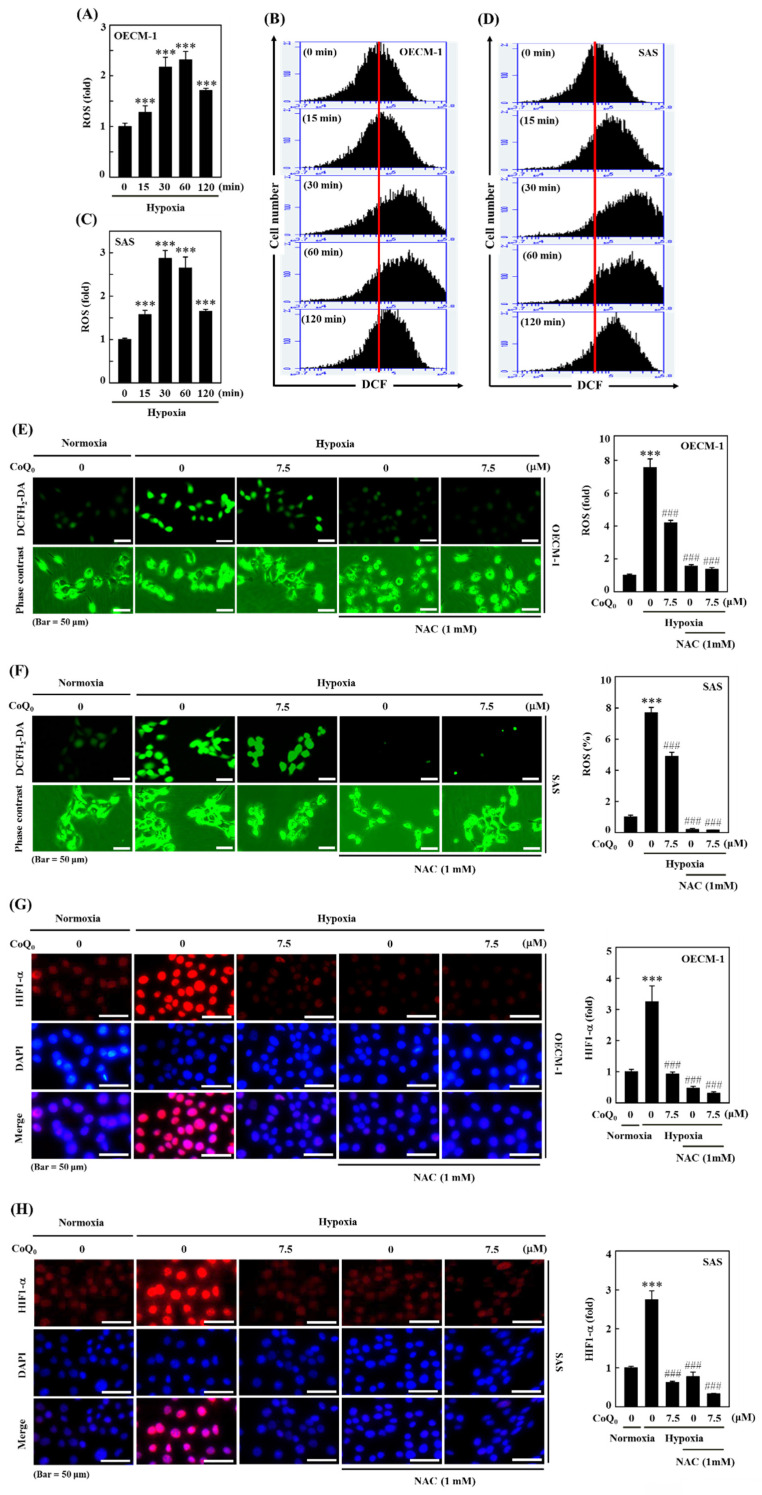
CoQ_0_ repressed hypoxia-mediated ROS and HIF-1α expression in SAS and OECM-1 cells. **(A-D)** The time-dependent effect on hypoxia-enhanced ROS generation as determined by measuring the fluorescence intensity of DCFH_2_-DA by flow cytometry. OECM-1 and SAS cells were treated with CoQ_0_ (7.5 μM) for 0-120 min under hypoxic conditions. **(E-F)** CoQ_0_ and/or NAC suppressed hypoxia-enhanced ROS generation, as shown by the fluorescence intensity of DCFH_2_-DA under a fluorescence microscope (Olympus Soft Imaging Solution). OECM-1 and SAS cells under hypoxia were exposed to CoQ_0_ (7.5 μM) or NAC (1 mM) for 60 or 30 min. **(G-H)** CoQ_0_ and/or NAC inhibited hypoxia-induced ROS-mediated HIF-1α expression as determined by using an immunofluorescence assay. OECM-1 and SAS cells under hypoxia were treated with CoQ_0_ (7.5 μM) or NAC (1 mM) for 24 h. Values are the mean ± SD (n=3). ***p < 0.001 compared with untreated cells. ^###^*p* < 0.001 compared with normoxia-treated cells.

The ROS production is well known to enhance HIF-1α expression. The efficacy of CoQ_0_ on hypoxia-induced intracellular ROS generation in OECM-1 and SAS cells was determined. As shown by the fluorescence microscopy and flow cytometry DCF assays, OECM-1 and SAS cells exposed to hypoxia produced a remarkable amount of ROS, which peaked at 30 min and 60 min (**Figure 2A-D**). Next, to assess whether the effect of CoQ_0_ treatment is mediated via an anti-oxidant pathway, we used N-acetylcysteine (NAC), a well-known anti-oxidant compound, for *in vitro* assays. Our results showed that pretreatment with CoQ_0_ (7.5 µM) and/or NAC (1 mM) notably suppressed hypoxia-triggered ROS generation in OECM-1 and SAS cells (**Figure 2E-F**). The combined effects of CoQ_0_ and NAC were similar to NAC alone treatment, whereas, there was a synergistic effect when CoQ_0_ and NAC were compared to CoQ_0_ alone, suggesting that CoQ_0_ follows an anti-oxidant mediated pathway. These data confirmed that exposure to hypoxia leads to the substantial release of ROS by OECM-1 and SAS cells, which was significantly abolished by CoQ_0_ and/or NAC treatment.

### CoQ_0_ inhibits hypoxia-induced ROS-mediated HIF-1α expression in OECM-1 and SAS cells

Hypoxia, which acts through ROS delivery and HIF-1α stabilization, is a key determinant of human tumor progression. To further reaffirm the action of CoQ_0_ on ROS-mediated HIF-1α expression under hypoxic conditions, we examined whether treatment with CoQ_0_ could alter HIF-1α expression. The immunofluorescence data demonstrated that CoQ_0_ (7.5 µM for 24 h) and/or NAC (1 mM for 24 h) treatment suppressed HIF-1α expression under hypoxia in OECM-1 and SAS cells (**Figure 2G-H**). These data confirmed that CoQ_0_ inhibited hypoxia-triggered ROS-mediated HIF-1α expression in HNSCC cells.

### CoQ_0_ suppresses the NLRP3/NFκB inflammasome in OECM-1 and SAS cells

**Figure 3:**
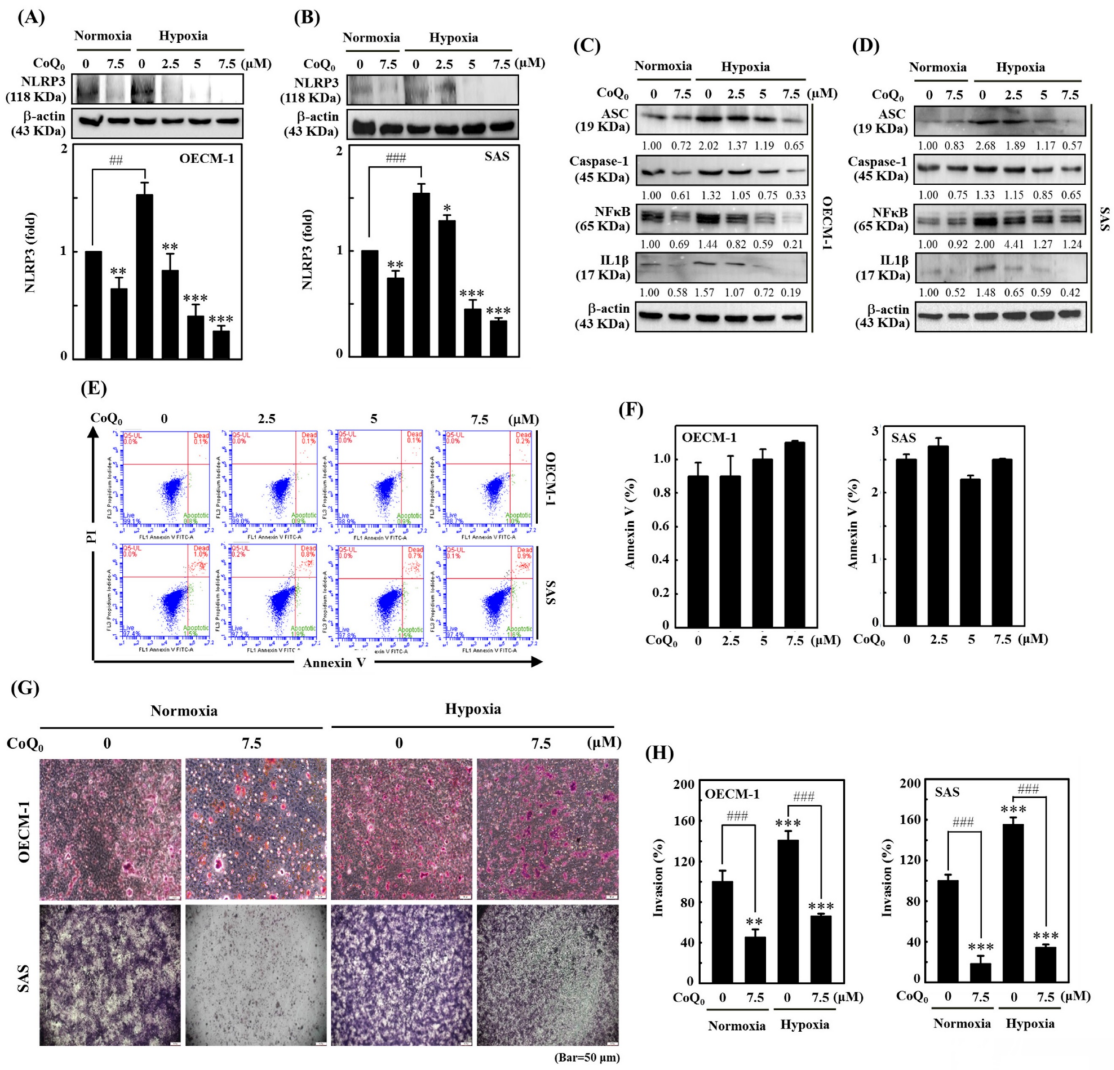
CoQ_0_ inhibits the NLRP3 inflammasome and invasion in OECM-1 and SAS cells under normoxia and hypoxia. Cells were incubated with CoQ_0_ (0-7.5 μM) for 24 h. **(A-B)** NLRP3 expression was determined by immunoblotting analysis. The protein densitometric quantifications were performed using commercially accessible software (AlphaEase, Genetic Technology Inc., Miami, FL, USA). **(C-D)** Western blotting shows IL-1β, ASC, Caspase-1, and NFκB expression. **(E-F)** Annexin V-FITC-negative cells analyzed by flow cytometry indicated normal cells.** (G-H)** Invasion was measured using BD Matrigel chambers. Values are the mean ± SD (n=3). **p < 0.01; ***p < 0.001 compared with untreated cells. ^###^*p* < 0.01 compared with normoxia-treated or untreated cells.

The NLRP3 inflammasome consists of the adaptor and effector proteins NLRP3, Caspase-1, and ASC, which are abundantly produced by cancer cells 32. Our data indicated that CoQ_0_ treatment (0-7.5 µM for 24 h) markedly suppressed NLRP3 expression in OECM-1 and SAS cells under normoxia and hypoxia (**Figure 3A-B**). Furthermore, the data showed that CoQ_0_ exposure inhibited ASC and caspase-1 expression in OECM-1 and SAS cells under normoxia and hypoxia (**Figure 3C-D**). NLRP3 and NFκB perform crucial functions in the regulation of inflammasome formation and the expression of IL-1β. The data showed that CoQ_0_ exposure inhibited NFκB and IL-1β in SAS and OECM-1 cells under normoxia and hypoxia (**Figure 3C-D**). Together, these results suggest that CoQ_0_ attenuates NLRP3 inflammation in HNSCC (OECM-1/SAS) cells under normoxia and hypoxia.

### CoQ_0_ inhibits the invasion and migration of OECM-1 and SAS cells

**Figure 4:**
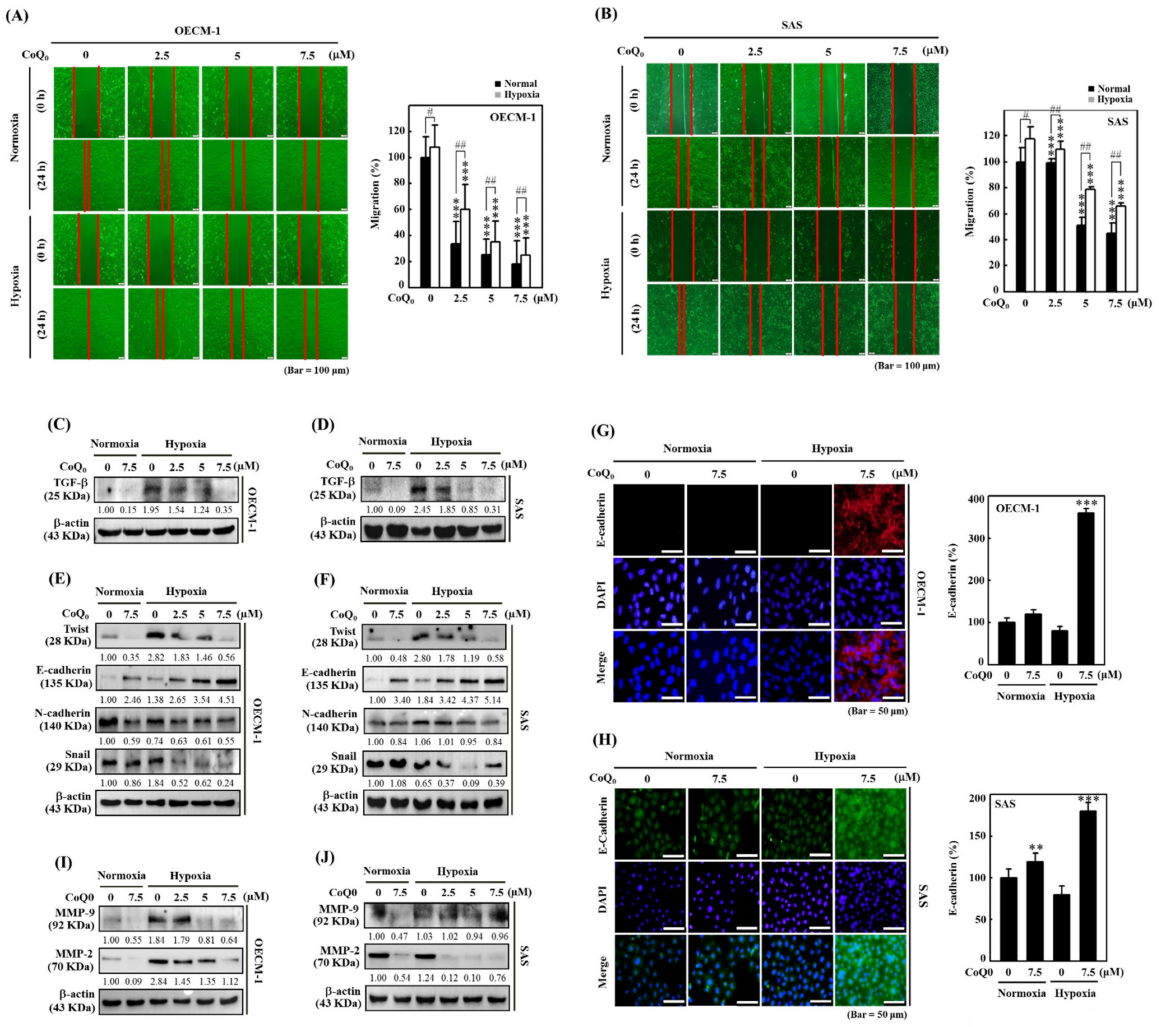
CoQ_0_ attenuated the migration and EMT in OECM-1 and SAS cells under normoxia and hypoxia. Cells were treated with CoQ_0_ (0-7.5 μM) for 24 h. **(A-B)** Cell migration in a wound-healing assay was observed by an optical microscope, and commercially available software was used to determine the wound healing area. **(C-D)** Cell migration (%) was calculated using commercially available software (Image-Pro Plus). **(E-F)** TGF-β expression was examined by immunoblotting analysis.** (G-H)** Immunoblot showing the protein expression of Twist, E-cadherin, N-cadherin, and Snail. **(I-J)** Immunofluorescence analysis of E-cadherin expression. **(K-L)** MMP-9 and MMP-2 expression were determined by immunoblotting. Values are the mean ± SD (n=3). **p < 0.01; ***p < 0.001 compared with untreated cells. ^#^*p* < 0.05; ^##^*p* < 0.01 compared with normoxia-treated cells.

Cancer cell invasion or migration impacts the early stages of metastasis and promotes the advancement of untreatable complex metastasis. We elucidated the impact of CoQ_0_ (0-7.5 μM for 24 h) on apoptosis. Flow cytometry analysis using Annexin V-FITC staining revealed that treatment of OECM-1 and SAS cells with CoQ_0_ caused no significant increase in the proportion of apoptotic cells (**Figure 3E-F**). These data indicated that treatment with 0-7.5 μM CoQ_0_ for 24 h had no cytotoxic effects on OECM-1 and SAS cells. Next, we assessed the impact of CoQ_0_ treatment on the invasion of OECM-1 and SAS cells. The data indicated that the invasion of OECM-1 and SAS cells under hypoxic conditions was more intensified than that under normoxia (**Figure 3G-H**). CoQ_0_ treatment conspicuously decreased OECM-1 and SAS cell invasion under normoxic and hypoxic conditions (**Figure 3G-H**). Furthermore, these data also revealed that the migration ability of OECM-1 and SAS cells under hypoxia was more prominent than that under normoxia; however, treatment with CoQ_0_ dose-dependently inhibited OECM-1 and SAS cell migration under normoxic and hypoxic conditions (**Figure 4A-B**).

### CoQ_0_ suppresses TGF-β expression in SAS and OECM-1 cells

TGF-β is known to promote EMT through the TGF-β/Smad/Snail molecular pathways 33. We examined whether CoQ_0_ (0-7.5 µM for 24 h) treatment could inhibit TGF-β in OECM-1 and SAS cells under normoxia and hypoxia. These data showed that TGF-β expression was more prominent under hypoxic conditions than that under normoxic conditions; however, exposure to CoQ_0_ significantly decreased TGF-β expression under both normoxia and hypoxia (**Figure 4A-D**).

### CoQ_0_ reverses EMT by decreasing Twist, Snail, and N-cadherin expression and enhancing E-cadherin expression in SAS and OECM-1 cells

EMT is a dynamic pathway that regulates cancer cell propensity by changing the mesenchymal markers Twist, N-cadherin, and Snail expression, and the epithelial marker E-cadherin expression 34. Western blotting showed that Twist, N-cadherin, and Snail expression in SAS and OECM-1 cells was higher under hypoxic conditions than under normoxic conditions (**Figure 4E-F**). However, treatment with CoQ_0_ decreased Twist, N-cadherin, and Snail expression (**Figure 4E-F**). In addition, Western blotting (**Figure 4E-F**) and immunofluorescence staining (**Figure 4G-H**) revealed that E-cadherin expression was lower under hypoxic conditions than under normoxic conditions, while CoQ_0_ treatment increased E-cadherin expression in OECM-1 and SAS cells under normoxia and hypoxia.

### CoQ_0_ inhibits MMP-2 and MMP-9 expression in OECM-1 and SAS cells

MMP-9 and MMP-2 are the principal proteolytic enzymes that promote cancer cell invasion by decomposing the basement membrane and then triggering cancer metastasis 35. The data indicated that the expression of MMP-2 and MMP-9 was higher under hypoxic conditions than under normoxic conditions, while exposure to CoQ_0_ inhibited MMP-9 and MMP-2 expression in OECM-1 and SAS cells (**Figure 4I-J**).

### CoQ_0_ decreases glucose absorption and lactate accumulation in OECM-1 and/or SAS cells

**Figure 5:**
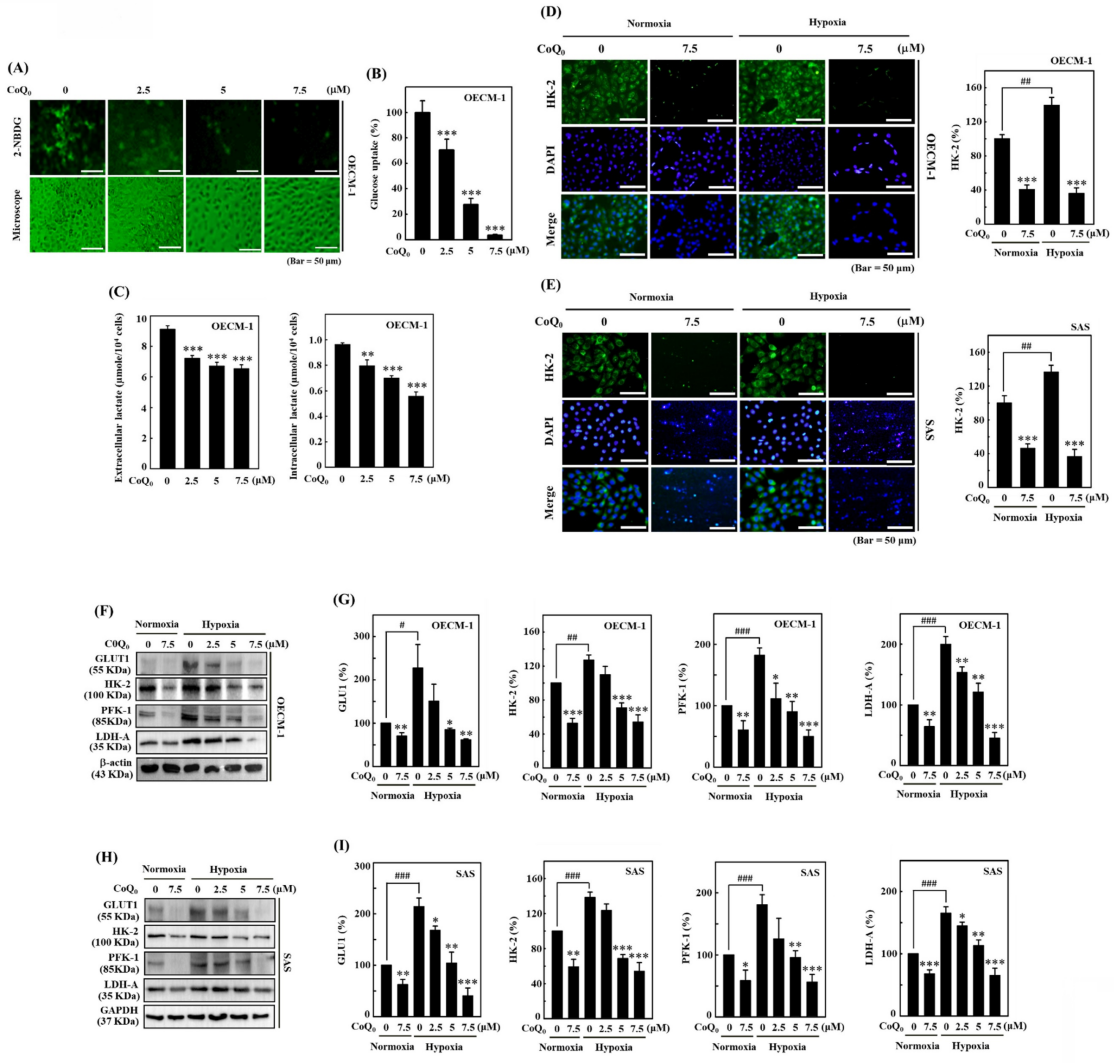
CoQ_0_ prevents glucose absorption/lactate accumulation and inhibits GLUT1/glycolytic enzyme (HK-2, PFK-1, and LDH-A) expression in OECM-1 and/or SAS cells. **(A-B)** Glucose uptake was examined under a fluorescence microscope. OECM-1 cells were treated with CoQ_0_ (0-7.5 μM) for 6 h, followed by incubation with 2-NBDG (100 μM). **(C)** Extracellular and intracellular lactate accumulation was examined by using a Cayman Lactate test kit. OECM-1 cells were treated with CoQ_0_ (0-7.5 μM) for 24 h. **(D-E)** The expression of the glycolytic enzyme HK-2 was examined using immunofluorescence analysis. Cells were exposed to CoQ_0_ (0 or 7.5 μM for 24 h) under normoxia and hypoxia. **(F-I)** Western blotting was used to examine GLUT1, HK-2, PFK-1, and LDH-A expression in OECM-1 and SAS cells. Cells were treated with CoQ_0_ (0-7.5 μM) for 24 h under normoxia and hypoxia. Values are the mean ± SD (n=3). *p < 0.05; **p < 0.01; ***p < 0.001 compared with untreated cells. ^#^*p* < 0.05; ^###^*p* < 0.001 compared with normoxia-treated cells.

The study used a noncytotoxic concentration of CoQ_0_ (0-7.5 μM for 24 h) in subsequent experiments to investigate its effect on the Warburg effect, which prefers aerobic glycolysis over mitochondrial oxidative respiration. An increase in glucose absorption and a preference for lactate accumulation in spite of the presence of oxygen are characteristics of the Warburg effect. Lactate, which is one of the products of glycolysis, has the capacity to change the cancer microenvironment and promote tumor development 36. A novel fluorescent glucose analog probe called 2-NBDG has been utilized extensively as a tracer for monitoring of glucose transport directly in a variety of cell types. In mammalian cells, it has been confirmed that 2-NBDG is intracellularly transported via the same glucose transporters (GLUTs) as glucose 37. Therefore, we used 2-NBDG, to assess glucose uptake in HNSCC cells. The data showed that CoQ_0_ treatment decreased aerobic glycolysis by suppressing glucose uptake and reducing extracellular and intracellular lactate accumulation in OECM-1 cells (**Figure 5A-C**).

The initial phase in the glucose metabolic pathway (aerobic glycolysis) involves the phosphorylation of Hexokinase (HK) 36. Immunofluorescence data showed that HK-2 expression under hypoxia was higher than that under normoxia, while CoQ_0_ treatment diminished HK-2 expression in OECM-1 and SAS cells (**Figure 5D-E**). These findings indicated that CoQ_0_ treatment attenuated the Warburg effect in HNSCC cells.

### CoQ_0_ decreases the expression of the glucose transporter GLUT1 and glycolytic enzymes HK-2, PFK-1, and LDH-A in OECM-1 and SAS cells

The glucose transporter GLUT1 facilitates the passage of glucose across cell membranes. PFK-1, which is an essential enzyme for aerobic glycolysis, catalyzes the transformation of fructose-6-phosphate and ATP into fructose-1,6-bisphosphate (FBP) and ADP 36. Pyruvate and L-lactate are transformed into each other by LDH-A. Our findings revealed that GLUT1, HK-2, PFK-1, and LDH-A protein expression were enhanced in OECM-1 and SAS cells under hypoxic conditions compared to normoxic conditions. However, CoQ_0_ (0-7.5 μM for 24 h) treatment suppressed GLUT1, HK-2, PFK-1, and LDH-A expression in OECM-1 and SAS cells under hypoxic and normoxic conditions in a dose-dependent manner (**Figure 5F-I**). Our results showed that CoQ_0_ inhibited HIF-1α-regulated glycolysis enzymes in SAS and OECM-1 cells under hypoxia and normoxia.

### CoQ_0_ suppresses the extracellular acidification rate (ECAR) and glycolytic intermediate levels in OECM-1 cells

The extracellular acidification rate (ECAR) is typically used to examine aerobic glycolysis 38.

Oligomycin is an ATP synthase (complex V) inhibitor which decreases electron flow across the electron transport chain, which in turn lowers cellular ATP generation and mitochondrial respiration or oxygen consumption rate (OCR). This causes the generation of energy to switch to glycolysis, and the maximum glycolytic capacity of the cell is revealed by the increase in ECAR that follows. The last injection with 2-DG (a glucose analog), competitively inhibits glycolysis through binding to glucose hexokinase, the initial enzyme in the glycolytic pathway. The subsequent drop in ECAR demonstrates that the ECAR produced in the experiment is due to glycolysis. The glycolytic capacity and glycolysis rate differences are indicative of the glycolytic reserve of the cells 39.

**Figure 6:**
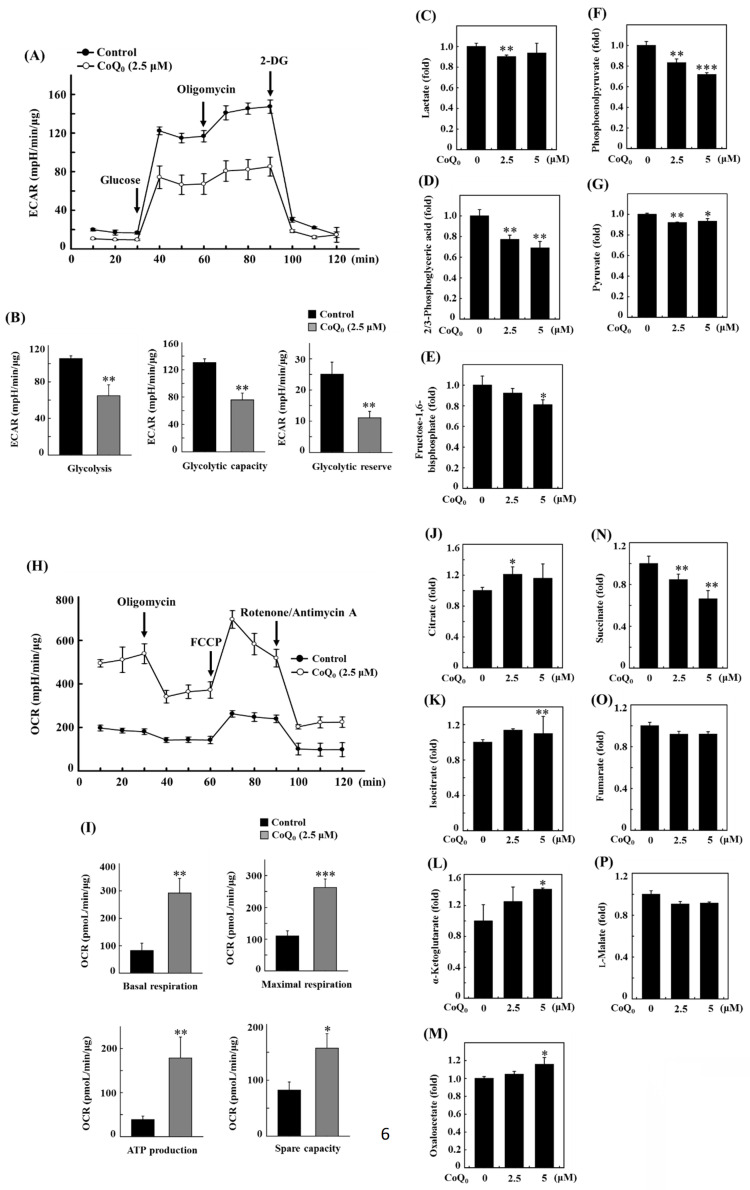
CoQ_0_ attenuated the extracellular acidification rate (ECAR)/aerobic glycolysis intermediates and augmented the oxygen consumption rate (OCR)/TCA cycle (mitochondrial oxidative phosphorylation) metabolites in OECM-1 cells. **(A-B)** The ECAR, glycolytic capacity, glycolysis, and glycolytic reserve were examined. Cells were treated with CoQ_0_ (0 or 2.5 μM) for 24 h. **(C-G)** Levels of aerobic glycolysis intermediates, including lactate **(C)**, 2/3-phosphoglycerate (2/3-PG)** (D)**, fructose 1,6-bisphosphate (FBP)** (E)**, phosphoenolpyruvate (PEP)** (F)**, and pyruvate **(G)**, were examined using an LC-ESI-MS metabolomics platform. Cells were treated with CoQ_0_ (0-5 μM) for 24 h. **(H-I)** The OCR, basal respiration, ATP generation, maximal respiration, and spare capacity were determined. Cells were treated with CoQ_0_ (0 or 2.5 μM) for 24 h.** (J-P)** Levels of TCA cycle metabolites, including citrate **(J)**, isocitrate **(K)**, α-ketoglutarate **(L)**, oxaloacetate **(M)**, succinate **(N)**, fumarate **(O)**, and L-malate **(P)**, were examined using an LC-ESI-MS metabolomics platform. Cells were treated with CoQ_0_ (0-5 μM) for 24 h. Values are the mean ± SD (n=3). *p < 0.05; **p < 0.01; ***p < 0.001 compared with untreated cells.

Measuring the efficacy of CoQ_0_ (0 and 2.5 μM for 24 h) on the ECAR allowed us to determine whether the Warburg effect was inhibited. The results disclosed that CoQ_0_ inhibited the glucose-stimulated ECAR and reduced the glycolytic reserve, glycolytic capability, and glycolysis in OECM-1 cells (**Figure 6A-B**). Furthermore, glycolytic intermediates were examined using LC-ESI-MS metabolomic analysis. The data showed that CoQ_0_ (0-5 μM for 24 h) treatment markedly reduced the lactate, 2/3-phosphoglycerate (2/3-PG), FBP, phosphoenolpyruvate (PEP), and pyruvate levels in OECM-1 cells (**Figure 6C-G**).

### CoQ_0_ increases the oxygen consumption rate (OCR) and TCA cycle (mitochondrial oxidative phosphorylation)-derived metabolite levels in OECM-1 cells

To further inspect the role of CoQ_0_ in the Warburg effect, we determined the OCR and measured TCA cycle-derived metabolite levels in OECM-1 cells. The results revealed that CoQ_0_ (0 and 2.5 μM for 24 h) treatment promoted the OCR and increased ATP production, basal respiration, maximal respiration, and spare capacity compared to nontreated OECM-1 cells (**Figure 6H-I**). Next, OECM-1 cells were incubated with CoQ_0_ (0-5 μM for 24 h), and then TCA cycle metabolite levels were tracked. LC-ESI-MS metabolomic analysis was used to measure the amounts of isocitrate, α-ketoglutarate, citrate, oxaloacetate, succinate, fumarate, and L-malate. There was an elevation in the amounts of citrate, isocitrate, α-ketoglutarate, and oxaloacetate in CoQ_0_-treated versus nontreated OECM-1 cells (**Figure 6J-M**). These metabolites, whose levels are increased by the Warburg effect, are consistent with enhancement in the TCA cycle (mitochondrial oxidative respiration). Nevertheless, there was no increase in the succinate, fumarate, or L-malate levels in CoQ_0_-treated OECM-1 cells (**Figure 6N-P**).

### CoQ_0_ ameliorates the Warburg effect in OECM-1 cells treated with the hypoxia-mimetic agent CoCl_2_

**Figure 7:**
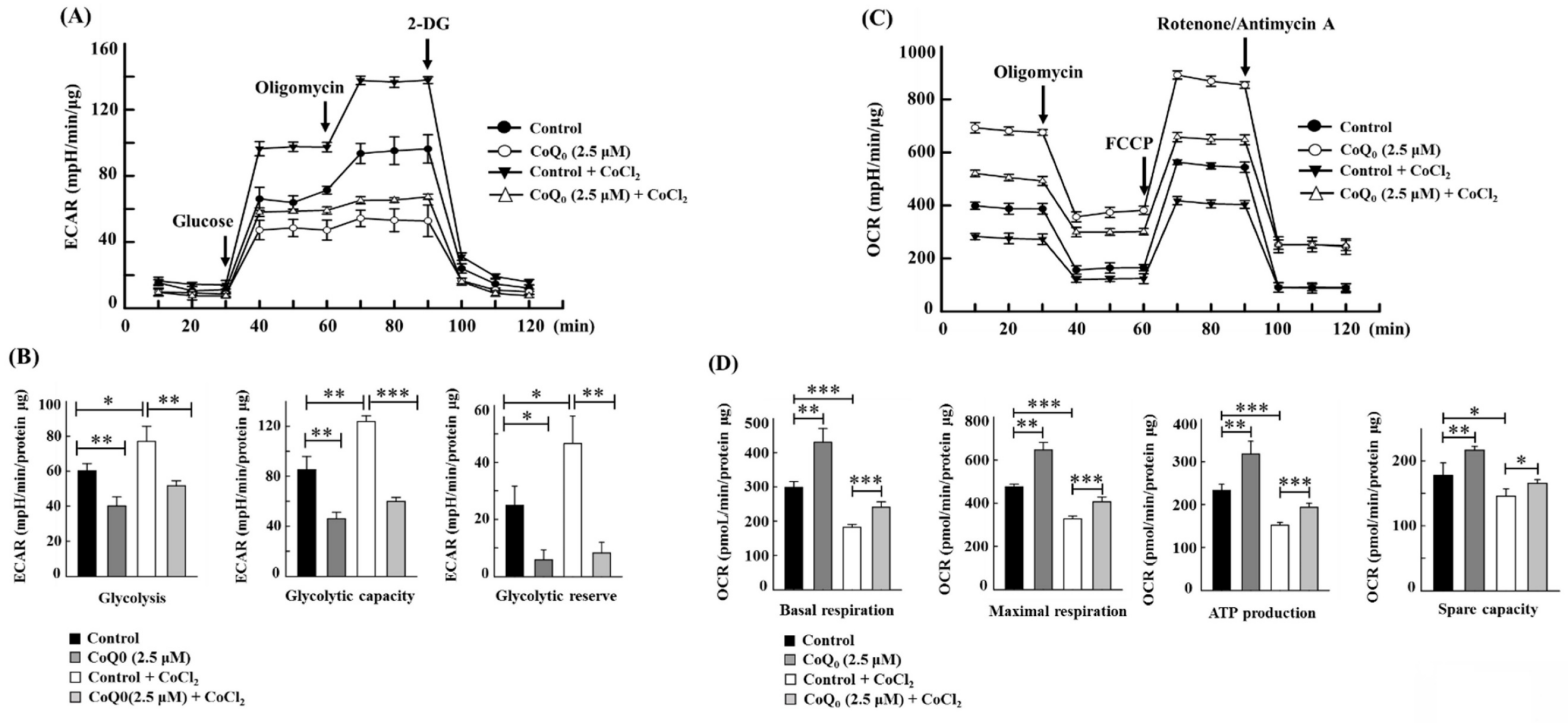
CoQ_0_ attenuated the ECAR and enhanced the OCR in OECM-1 cells exposed to normoxia and the hypoxia-mimetic agent CoCl_2_. Cells were incubated with CoQ_0_ (0 or 2.5 μM for 24 h) under normoxia and hypoxia (CoCl_2_, 10 μM). **(A-B)** The ECAR, glycolytic capacity, glycolysis, and glycolytic reserve were increased by CoCl_2_ and attenuated by CoQ_0_ treatment. **(C-D)** The OCR, basal respiration, ATP synthesis, maximum respiration, and spare capacity were inhibited by CoCl_2_ and enhanced by CoQ_0_ treatment. Values are the mean ± SD (n=3). *p < 0.05; **p < 0.01; ***p < 0.001 compared with untreated or CoCl_2_-treated cells.

CoCl_2_ is a commonly used chemical that artificially induces hypoxia, leading to the stabilization of HIF-1α 40. As expected, CoCl_2_ treatment resulted in hypoxia in OECM-1 cells, leading to an increased ECAR and a decreased OCR (**Figures 7A, and 7C**). Compared with non-CoCl_2_ treatment, the increase in the ECAR and decrease in the OCR were more pronounced after CoCl_2_ treatment (**Figures 7A, and 7C**). However, in both CoCl_2_-stimulated and control OECM-1 cells, we discovered that CoQ_0_ (0 and 2.5 μM for 24 h) inhibits the glucose-stimulated ECAR and suppresses glycolysis, glycolytic capability, and glycolytic reserves (**Figure 7A-B**). Additionally, the elevation in glycolysis, glycolytic capability, and glycolytic reserves in the CoCl_2_ treatment group were more pronounced than those in the non-CoCl_2_ treatment group (**Figure 7B**). In addition, treatment with CoQ_0_ (0 and 2.5 μM for 24 h) increased the OCR, basal respiration, ATP generation, maximal respiration, and spare capacity compared to OECM-1 cells treated without or with CoCl_2_ (**Figure 7C-D**). Moreover, the increases in basal respiration, ATP generation, maximal respiration, and spare capacity in non-CoCl_2_-treated cells were more prominent than those in CoCl_2_-treated OECM-1 cells (**Figure 7D**). These findings demonstrated that CoQ_0_ suppresses the ECAR (Warburg effects) and enhances the OCR (mitochondrial oxidative phosphorylation) under both normoxia and hypoxia (CoCl_2_).

### Effect of HIF-1α knockdown in OECM-1 and SAS cells under hypoxia

**Figure 8:**
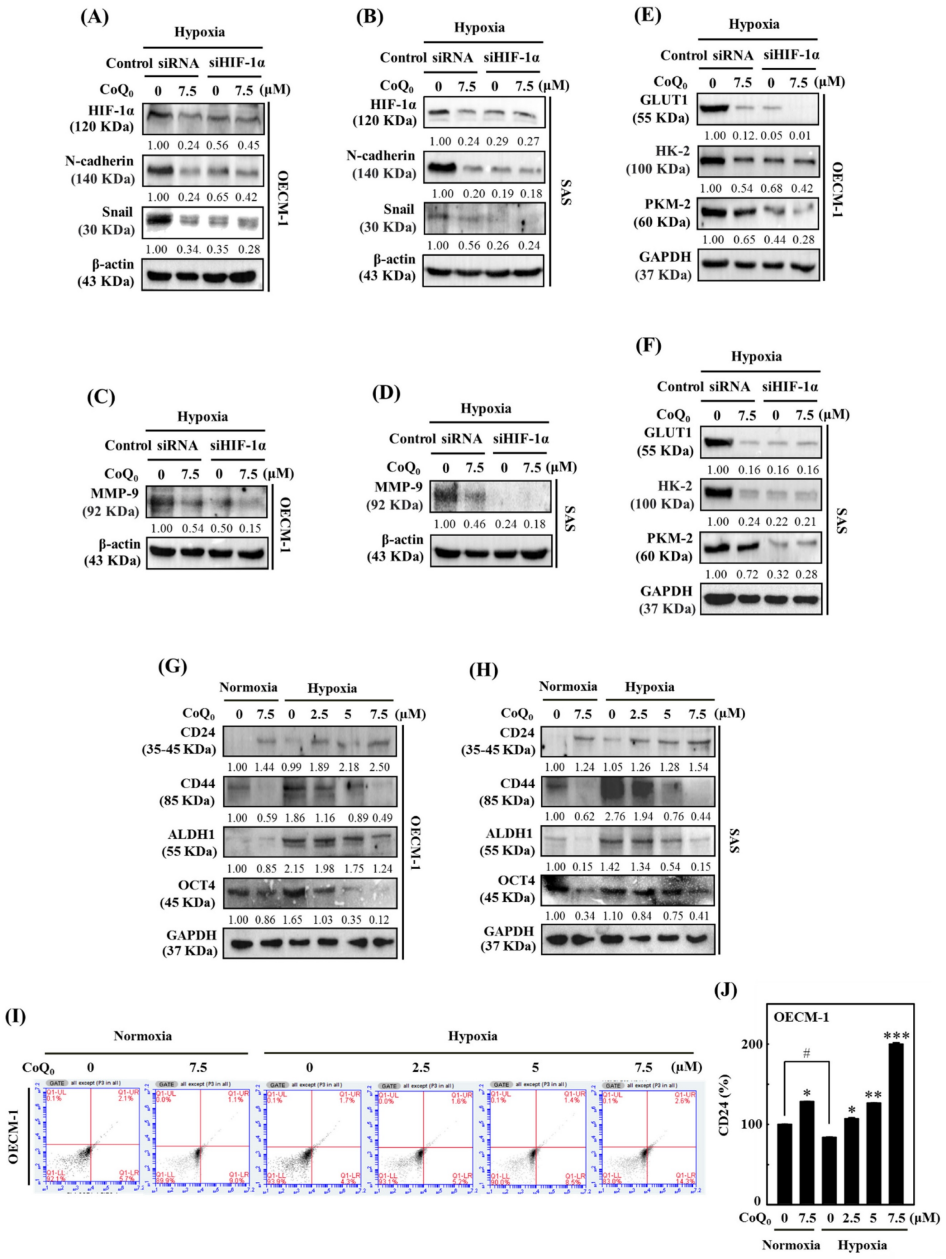
HIF-1α silencing reversed CoQ_0_-mediated anti-metastasis and metabolic reprogramming, and CoQ_0_ prevented cancer stem-like characteristics in OECM-1 and/or SAS cells under normoxia and/or hypoxia. **(A-F)** Cells were transfected with siRNAs targeting HIF-1α or nontargeting control siRNAs. Next, the cells were incubated with CoQ_0_ (7.5 µM) for 24 h under hypoxic conditions. **(A-B)** HIF-1α, N-cadherin, and Snail expression were determined using immunoblotting analysis. **(C-D)** Western blotting analysis shows MMP-9 expression. **(E-F)** GLUT1, HK-2, and PKM-2 expression were assessed using immunoblotting analysis. **(G-J)** Cells were treated with CoQ_0_ (0-7.5 μM for 24 h) under normoxia and/or hypoxia. **(G-H)** Expression of the stem cell markers CD24, CD44, ALDH1, and OCT4 in OECM-1 and SAS cells was determined by immunoblotting analysis. **(I-J)** CD24 expression in OECM-1 cells was assessed by flow cytometry analysis. Values are the mean ± SD (n=3). *p < 0.05; **p < 0.01; ***p < 0.001 compared with untreated cells. ^#^*p* < 0.05 compared with normoxia-treated cells.

To further verify the impact of HIF-1α in HNSCC cells under hypoxia (1% O_2_), siRNAs specifically targeting HIF-1α expression were transfected into OECM-1 and SAS cells. Silencing HIF-1α further enhanced CoQ_0_-suppressed N-cadherin and Snail expression, suggesting a major contribution of HIF-1α inhibition to CoQ_0_-triggered anti-EMT effects in OECM-1 and SAS cells under hypoxic conditions (**Figure 8A-B**). As above, a combinatorial effect of CoQ_0_ and HIF-1α knockdown was observed on MMP-9 expression, which was decreased compared to hypoxia alone-treated OECM-1 and SAS cells (**Figure 8C-D**). Furthermore, as shown in **Figure 8E-F**, siHIF-1α enhanced the effect of CoQ_0_ and additionally inhibited GLUT1, HK-2, and PKM-2 expression, confirming the crucial contribution of HIF-1α inhibition in the CoQ_0_-attenuated Warburg effect under hypoxic conditions in OECM-1 and SAS cells. Together, these findings suggested that silencing HIF-1α expression exerted a combinatorial effect with CoQ_0_-mediated inhibition of N-cadherin, Snail, MMP-9, GLUT1, HK-2, and PKM-2 expression, suggesting an important role of HIF-1α inhibition in the CoQ_0_-mediated anti-metastasis effects and metabolic reprogramming of OECM-1 and SAS cells under hypoxic conditions.

### CoQ_0_ upregulates CD24 expression and downregulates CD44 expression in OECM-1 and SAS cells

Cancer stem cells are unique in their ability to undergo EMT, differentiation, and self-renewal, and they exhibit CD24^low^ and CD44^high^ expression 41. The results showed that CoQ_0_ (0-7.5 µM for 24 h) treatment increased CD24 and decreased CD44 expression in OECM-1 and SAS cells under normoxia and hypoxia (**Figure 8G-H**). Additionally, we determined whether CoQ_0_ has an impact on ALDH1 and OCT4, which are markers of cancer stem cells. Similar results were observed for ALDH1 and OCT4 expression, which was decreased by CoQ_0_ (**Figure 8G-H**). Flow cytometry analysis further confirmed that CoQ_0_ increases CD24 expression in OECM-1 cells under normoxic and hypoxic conditions (**Figure 8I-J**). Consistent with findings from other studies, we found decreased expression of the cancer stem cell markers CD44, ALDH1, and OCT4 in OECM-1 and SAS cells under hypoxic conditions compared to normoxic conditions.

### CoQ_0_ reverses IL-6-stimulated morphological changes (epithelial-to-fibroblastic phenotype) in salivary gland epithelial (SG) cells

**Figure 9:**
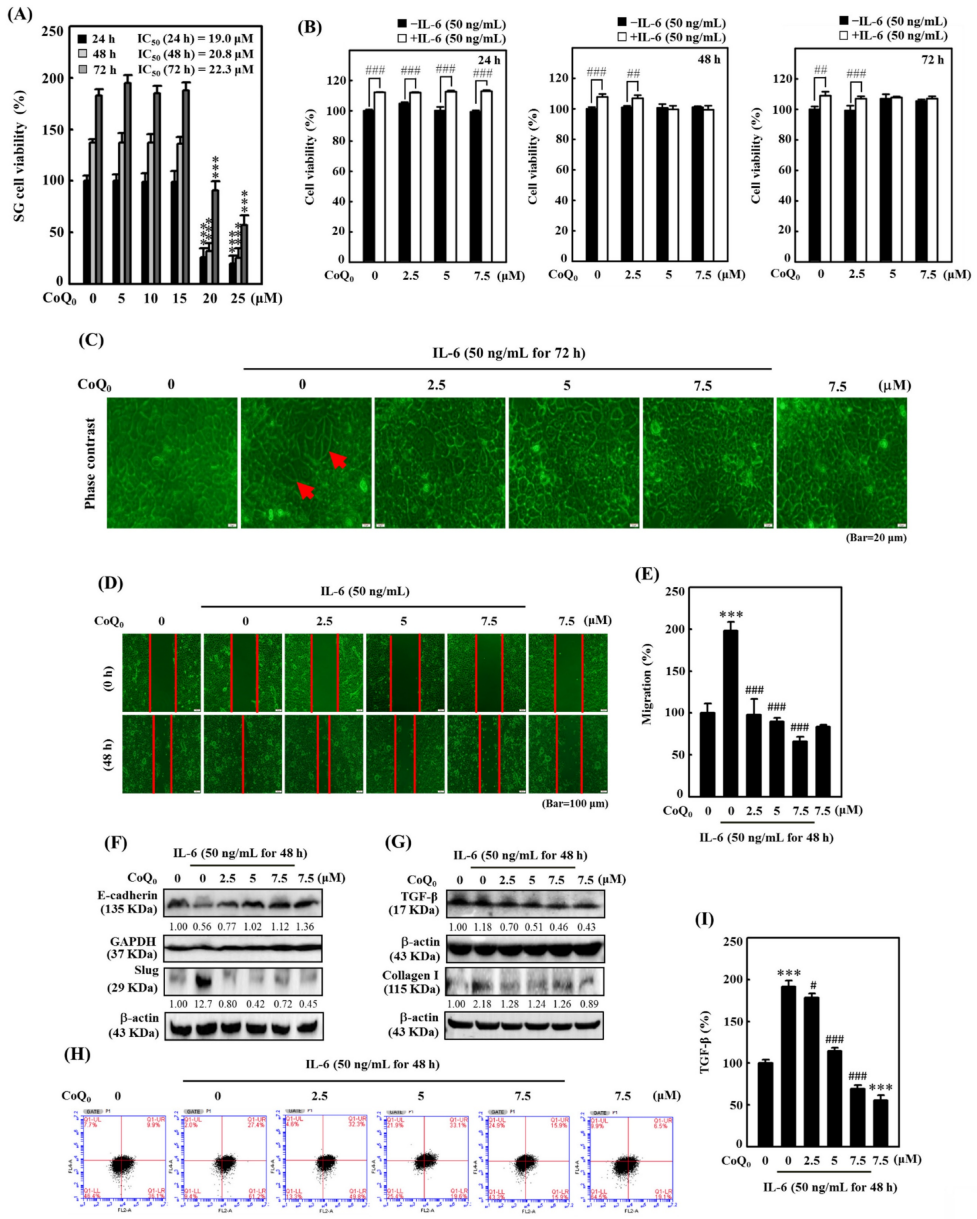
CoQ_0_ inhibited IL-6-stimulated morphological changes (epithelial-to-fibroblastic phenotype), metastasis/EMT, and fibrosis in human salivary gingival epithelial SG cells. **(A)** Cells (in medium supplemented with 1% FBS) were incubated with CoQ_0_ (0-25 μM) for 24, 48, or 72 h. The MTT colorimetric test was utilized to determine cell viability.** (B)** Cells (in medium supplemented with 1% FBS) were incubated with CoQ_0_ (0-7.5 μM) for 24, 48, or 72 h without or with IL-6 (50 ng/mL). Cell viability was assessed using the MTT colorimetric test.** (C)** The morphological changes were determined using a phase contrast microscope. Arrows indicate fibroblastic cells. Cells (medium supplemented with 1% FBS) were treated with CoQ_0_ (0-7.5 μM) for 72 h without or with IL-6 (50 ng/mL). **(D-I)** Cells were stimulated without or with IL-6 (50 ng/mL) following CoQ_0_ (0-7.5 μM for 48 h) treatment.** (D-E)** Cell migration in a wound-healing assay was observed by an optical microscope, and commercially available software was used to determine the wound healing area. **(F)** E-cadherin and Slug expression were determined by immunoblotting analysis. **(G)** TGF-β and Collagen I expression were determined by Western blotting. **(H-I)** TGF-β expression was examined by flow cytometry analysis. Values are the mean ± SD (n=3). ***p < 0.001 compared with untreated cells. ^#^*p* < 0.05; ^##^*p* < 0.01; ^###^*p* < 0.001 compared with IL-6-treated cells.

The overexpression of the cytokine IL-6 is known to promote EMT and fibrosis in several inflammatory conditions that predispose to cancer development 42. To determine the significance of CoQ_0_ in preventing EMT and fibrosis, we used IL-6-stimulated SG cells. We found that CoQ_0_ (0-25 µM for 24, 48, or 72 h) treatment markedly decreased the viability of SG cells, with IC_50_ values of 19.0, 20.8, or 22.3 µM, respectively (**Figure 9A**). Furthermore, the MTT of noncytotoxic CoQ_0_ concentrations (0-7.5 μM for 24, 48, or 72 h) on IL-6 (50 ng/mL)-stimulated SG cells were also determined. The data showed that IL-6 stimulation with CoQ_0_ treatment increased SG cell viability (**Figure 9B**). In addition, SG cells incubated with IL-6 (50 ng/mL for 72 h) exhibited abnormal morphological changes from an epithelial-to-fibroblastic phenotype (**Figure 9C**). However, CoQ_0_ (0-7.5 μM for 72 h) treatment reversed the IL-6-induced fibrotic effects and restored the fibroblastic-to-epithelial phenotype in SG cells (**Figure 9C**).

### CoQ_0_ inhibits IL-6-stimulated EMT in SG cells

We also examined the impact of CoQ_0_ (0-7.5 μM for 48 h) treatment on EMT and migration in SG cells. Our findings disclosed that CoQ_0_ significantly suppressed the migration of IL-6-stimulated SG cells (**Figure 9D-E**). Additionally, we assessed the E-cadherin (an epithelial phenotype marker) and Slug (a mesenchymal phenotype marker) protein expression, which are known to regulate the EMT process, in IL-6-stimulated SG cells. Compared to the control, CoQ_0_ treatment increased E-cadherin expression and decreased Slug expression in IL-6-stimulated SG cells in a dose-dependent manner (**Figure 9F**). These results revealed that CoQ_0_ treatment could have an anti-EMT effect on SG cells that had been incubated with IL-6.

### CoQ_0_ suppresses fibrosis by inhibiting TGF-β and Collagen I production in IL-6-stimulated SG cells

TGF-β plays a crucial role in myofibroblast phenoconversion, which is primarily responsible for fibrogenic effects. In addition, Collagen I is a key pathogenic factor in fibrotic conditions. We investigated the effect of CoQ_0_ treatment (0-7.5 μM for 48 h) on TGF-β and Collagen I expression in IL-6-stimulated (50 ng/mL for 48 h) SG cells. The western blot findings demonstrated that CoQ_0_ treatment diminished TGF-β and Collagen I expression in IL-6-stimulated SG cells in a dose-dependent manner (**Figure 9G**). TGF-β expression was further confirmed by flow cytometry analysis**,** which showed CoQ_0_ significantly decreased TGF-β expression in a dose-dependent manner in IL-6-stimulated SG cells (**Figure 9H-I**).

### CoQ_0_ suppresses the growth of OECM-1 xenograft tumors

**Figure 10:**
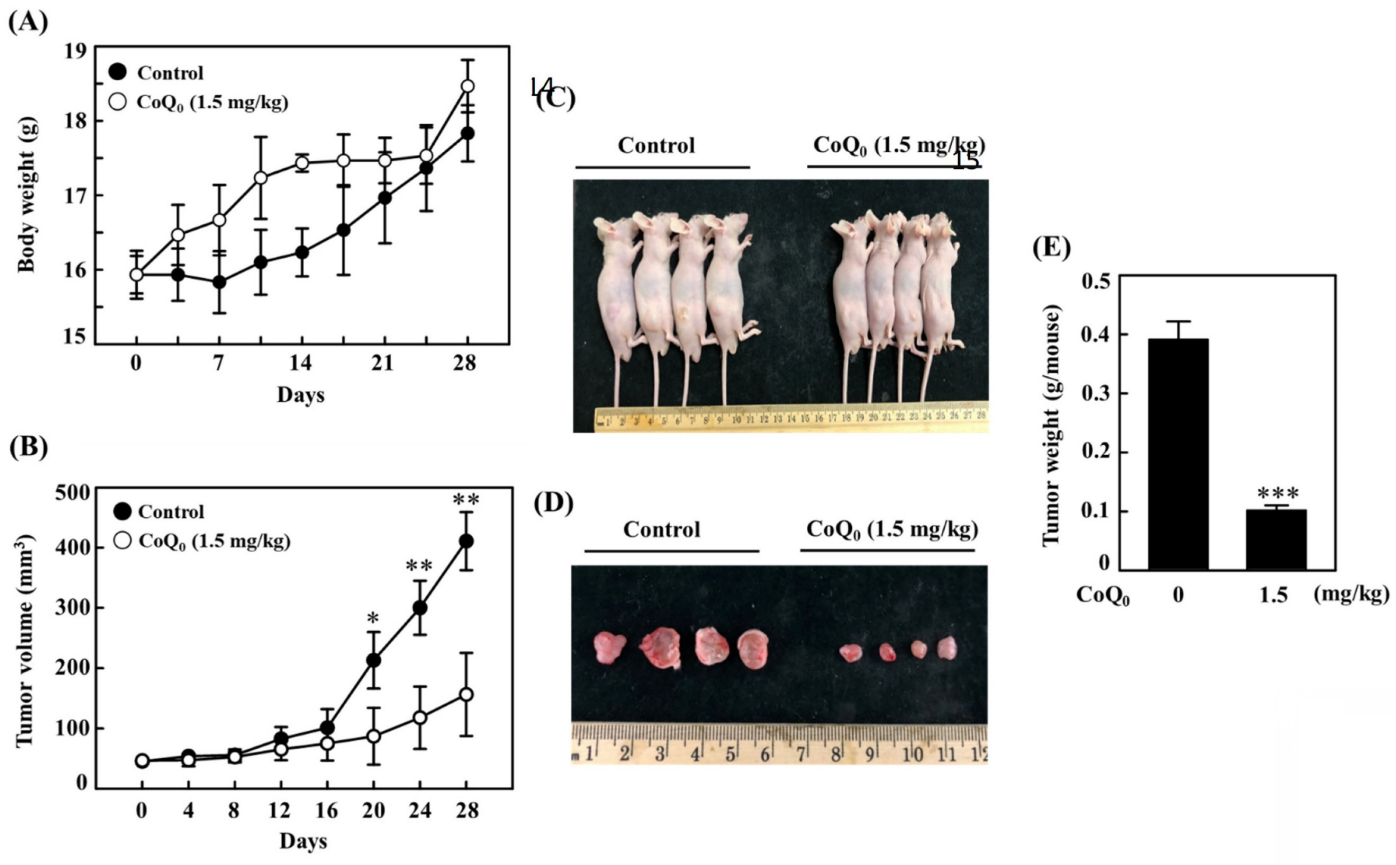
CoQ_0_ treatment inhibits OECM-1 xenograft tumors *in vivo.* Every two days for a total of 28 days, athymic nude mice were administered either 1.5 mg/kg CoQ_0_ or control (0.1% DMSO). **(A-B)** Tumor progression was assessed by evaluating body weight, which was measured twice every 7 days, and tumor volume, which was measured every 4 days. The animals were euthanized, and the tumor tissues were harvested and weighed on the 28^th^ day after OECM-1 cell implantation. **(C-D)** Images showing tumor sizes before and after dissection. **(E)** Tumor weight (g/mouse) is shown in the histogram. Values are the mean ± SD (n=3). *p < 0.05; **p < 0.01; ***p < 0.001 compared with untreated animals.

We investigated the anticancer potential of CoQ_0_ in a model of athymic nude mice with OECM-1 xenografts (**Figure 10A**). The data revealed that there was no apparent body weight reduction throughout CoQ_0_ therapy, and all the animals were healthy (**Figure 10A**). The tumor volume was significantly increased after OECM-1 cell inoculation into control nude mice. However, CoQ_0_ (1.5 mg/kg) treatment decreased the tumor volume in nude mice in a time-dependent manner (**Figure 10B**). Additionally, OECM-1-xenografted tumors were removed and weighed from each sacrificed mouse at the end of the 28-day period. The tumor mass in CoQ_0_-treated nude mice was reduced and circumscribed in comparison to the control group (**Figure 10C-E**). Moreover, we did not observe any overt toxicity (body weight and microscopic inspection of vital organs) in CoQ_0_-treated mice. Together, these results showed that tumor development in mice with OECM-1 xenografts was effectively inhibited by CoQ_0_ treatment.

## Discussion

Phytochemicals and traditional medicines are being investigated to reduce the gap associated with currently available cancer treatments 43. In this investigation, using noncytotoxic concentrations of CoQ_0_ (0-7.5 μM), the molecular mechanisms by which CoQ_0_ suppresses the NLRP3 inflammasome, metastasis/EMT, and the Warburg effect by inhibiting ROS-mediated HIF-1α expression in OECM-1 and SAS cells under normoxic and hypoxic conditions were elucidated. The tumor microenvironment, which is widely known to be a significant promoter of angiogenesis, features hypoxia as one of its primary properties. The hypoxic tumor microenvironment leads to HIF-1 activation via the assembly of the HIF-1α and HIF-1β subunits. Activated HIF-1 leads to angiogenesis and metabolic reprogramming in tumor cells. This study revealed more prominent HIF-1α expression in OECM-1 and SAS cells under hypoxia than under normoxia; however, treatment with CoQ_0_ inhibited HIF-1α expression under both normoxia and hypoxia (1% O_2_ or CoCl_2_). Previous studies have suggested pleiotropic effects of HIF-1α on several cellular and metabolic mechanisms that are related to tumorigenesis. Higher levels of HIF-1α expression are associated with the NLRP3 inflammasome, EMT/metastasis, and Warburg effect in tumor cells 31. Our findings supported the hypothesis that CoQ_0_ suppressed HIF-1α expression, which therefore suppressed the NLRP3 inflammasome, EMT/metastasis, and Warburg effect in HNSCC cells under normoxia and hypoxia.

Inflammasomes constitute an assembly of protein complexes comprised of several proteins, including NLRP3, which recognize many diverse inflammation-inducing stimuli such as stress, acute injury, microbial infections, etc. By directly activating caspase-1, inflammasomes trigger the release of effective pro-inflammatory cytokines. A recent study showed that periodontal pathogens may contribute to HNSCC pathogenesis by causing chronic inflammation and increasing NLRP3/IL-1β expression due to inflammasome dysregulation 44. Notably, periodontitis-associated bacteria may stimulate NLRP3 and enhance the progression and development of tumors in an *in situ* oral squamous cell carcinoma (OSCC) model in mice 45. In a carcinogen-induced OSCC model, the intracellular ROS induced by 5-FU enhanced the NLRP3 inflammasome and IL-1β expression, which in turn mediated the onset of chemoresistance and tumor cell proliferation 46. The downregulation of NLRP3 expression significantly decreased caspase-1 cleavage and IL-1β generation, whereas E-cadherin expression was enhanced in OSCC cells 47. Another study showed that inhibition of the NLRP3 inflammasome hampered the invasiveness of HNSCC cells 48. In addition, the instigation of the NLRP3 inflammasome by LPS and ATP upregulated cancer stem cell characteristics and induced sphere-forming and colony formation abilities 49. In the head and neck squamous cell carcinoma, treatment with the NLRP3 inhibitor MCC950 decreased sphere and colony formation as well as cancer stem cell marker BMI1, ALDH1, and CD44 expression 49. In an *in vitro* model employing OSCC cells and an *in vivo* xenograft model of oral cancer, an NLRP3 inflammasome inhibitor BAY-117082 significantly reduced NLRP3, ASC, caspase-1, IL-1β, and IL-18 expression and a reduction in tumor growth 50. In this study, we confirmed that the inflammatory NLRP3, ASC/caspase-1, and IL-1β expression, and EMT/metastasis was inhibited by CoQ_0_ under normoxia and hypoxia.

The overwhelming production of ROS eventually results in increased HIF-1α stabilization and expression. Previous research investigations have demonstrated that a functional mitochondrial electron transport chain is necessary for hypoxia-induced ROS release, which is crucial for HIF-1α stability 51. Following proline hydroxylation by prolyl hydroxylases, HIF-1α is damaged under normoxic conditions. Hypoxia prevents HIF-1α hydroxylation, which causes it to accumulate. Our findings revealed that CoQ_0_ and/or NAC treatment suppressed hypoxia-induced intracellular ROS generation in both OECM-1 and SAS cells. In addition, the current study also confirmed that pretreatment with CoQ_0_ and/or NAC inhibited hypoxia-induced HIF-1α expression in HNSCC cells. Our findings indicated that inhibition of hypoxia-induced ROS by CoQ_0_ strongly reduced HIF-1α expression, disrupting signaling related to the malignant growth of HNSCC cells. Our previous investigation showed that CoQ_0_ suppresses LPS-stimulated ROS-mediated inflammatory NFκB/AP-1 activation through antioxidant Nrf2 pathways in macrophages 17. Furthermore, CoQ_0_ inhibits LPS/ATP-stimulated ROS-mediated NLRP3 inflammasome via mitophagy induction and Nrf2 activation in macrophages 52. In addition, CoQ_0_ exerts anti-angiogenic effects via the inhibition of TNF-α-induced ROS-mediated NFκB activation and Nrf2 pathway activation in human endothelial cells 53. These investigations, along with our results (CoQ_0_ inhibits hypoxia-stimulated ROS-mediated HIF-1α expression), revealed the pharmacological activity of CoQ_0_. Therefore, it has been proposed that CoQ_0_ inhibits hypoxia-induced ROS-mediated HIF-1α expression and thus attenuates the NLRP3-mediated inflammation, EMT/metastasis, and Warburg effect in HNSCC cells, which have been suggested as possible targets for cancer therapy.

In this investigation, CoQ_0_ served as a mediator that prevented NLRP3 inflammasome activation in OECM-1 and SAS cells. When the NLRP3 inflammasome is activated, the adaptor protein ASC colocalizes with Caspase-1. Caspase-1 is a cysteine protease that cleaves pro-IL-1β to generate IL-1β, which leads to proinflammatory effects. There are currently no approved drugs that can prevent NLRP3 inflammasome activation, despite the findings that neutralization of IL-1β has anti-inflammatory effects 54. The data showed that CoQ_0_ suppresses the NLRP3 inflammasome via ASC, caspase-1, and IL-1β inhibition under normoxia and hypoxia in HNSCC cells.

The inhibition of hypoxia-induced HIF-1α suppresses the migration, invasion, and EMT of tumor cells 55. E-cadherin is an adherence junction protein that is essential for maintaining cell integrity and cellular communication. However, the loss of E-cadherin is related to EMT, which is necessary for the development of cancer metastasis. Hence, reversing or preventing the loss of E-cadherin may help to inhibit EMT and metastasis. The expression of N-cadherin, which is a cell surface mesenchymal marker, is commonly increased in cancer cells, which provides a mechanism for transendothelial migration 35. The study revealed that CoQ_0_ prevented migration and invasion and reversed EMT by upregulating E-cadherin and downregulating N-cadherin in OECM-1 and SAS cells under normoxia and hypoxia. Twist, which is an EMT modulator, is necessary for the development of HNSCC (OECM-1 and SAS) cells. In metastatic carcinomas, Twist is frequently overexpressed or its promoter is methylated. The mechanisms underlying EMT include activated Twist, which enhances N-cadherin and reduces E-cadherin expression. Twist expression is regulated by HIF-1α through a direct interaction with the hypoxia-response element in the proximal promoter 55. HIF-1α and Twist coexpression are linked to HNSCC metastasis and indicate a poor prognosis in patients 56. In many malignancies, EMT involves NFκB-mediated Snail expression, which promotes cell invasion and migration. Snail acts as a transcriptional repressor that directly inhibits E-cadherin expression 34. Matrix metalloproteinases (MMPs), which are important for the degradation of the cellular basement membrane, are critical players in invasion and migration. Twist has been associated with MMP activation 57. The data showed that CoQ_0_ inhibited EMT/metastasis by attenuating Twist, Snail, and MMP-9/-2 expression in OECM-1 and SAS cells under normoxia and hypoxia. In addition, HIF-1α knockdown exerted a combinatorial effect with CoQ_0,_ which enhanced the inhibition of N-Cadherin, Snail, and MMP-9 expression under hypoxia, confirming the hypothesis that CoQ_0_ exerted anti-metastasis/EMT effects via HIF-1α inhibition in HNSCC cells.

Next, we investigated whether CoQ_0_ modulated metabolic reprogramming and the Warburg effect in HNSCC cells. HIF-1α activation regulates critical enzymes that are associated with aerobic glycolysis, which alternately feed into the Warburg effect 58. We found that CoQ_0_ decreased glucose absorption and L-lactate accumulation in OECM-1 cells. An increase in glucose absorption and a preference for lactate accumulation in the presence of oxygen are indications of the Warburg effect 36. Lactate, which is one of the products of aerobic glycolysis, can alter the cancer microenvironment and impact tumor progression 36. The data confirmed that CoQ_0_ suppressed glucose uptake, lactate accumulation, GLUT1 expression, and the HIF-1α-regulated expression of glycolysis-related genes (HK-2, PFK-1, and LDH-A) in OECM-1 and SAS cells. Moreover, HIF-1α knockdown exerted a combinatorial effect with CoQ_0_, enhancing the inhibition of GLUT1 and HK-2 expression under hypoxic conditions, confirming the hypothesis that CoQ_0_ promoted metabolic reprogramming via HIF-1α inhibition in HNSCC cells. It is generally recognized that the Warburg effect is characterized by the dominance of aerobic glycolysis over mitochondrial oxidative phosphorylation. This study discovered that CoQ_0_ diminished the ECAR as well as the glycolytic capacity, glycolytic reserve, and glycolysis under normoxia and hypoxia (CoCl_2_) in OECM-1 cells. CoCl_2_ artificially induces hypoxia, leading to the stabilization of HIF-1α. LC-ESI-MS metabolomic analysis revealed that CoQ_0_ attenuated the Warburg effect and decreased the levels of the glycolytic intermediates lactate, 2/3-PG, FBP, PEP, and pyruvate. CoQ_0_, on the other hand, led to an increase in the OCR, ATP production, maximal respiration, basal respiration, and spare capacity in OECM-1 cells under normoxic and hypoxic conditions (CoCl_2_). After CoQ_0_ treatment, the levels of the TCA cycle intermediate isocitrate, citrate, α-ketoglutarate, and oxaloacetate were markedly enhanced. The increased levels of these TCA cycle metabolites are consistent with improvements in the Warburg effect. Our findings confirmed that CoQ_0_ altered the Warburg effect (improved mitochondrial oxidative phosphorylation and suppressed aerobic glycolysis) via HIF-1α inhibition under normoxia and hypoxia in HNSCC cells.

The sensor of hypoxia HIF-1α also regulates the characteristics of cancer stem cells, including their ability to undergo EMT, differentiation, and self-renewal. CD24^low^ and CD44^high^ expression are commonly associated with cancer stem cells 41. Our findings showed that CoQ_0_ upregulated CD24 expression and downregulated CD44 expression in a concentration-dependent manner, shifting the CD24/CD44 ratio in favor of suppressing the cancer stem-like properties of OECM-1 and SAS cells under normoxia and hypoxia. Similarly, CoQ_0_ reduced ALDH1 and OCT4 expression in OECM-1 and SAS cells. ALDH1 and OCT4 are markers of cancer stem cells that participate in the differentiation and self-renewal of tumor cells 59, 60. Our data demonstrated that CoQ_0_ treatment decreased ALDH1, and OCT4 prevented the EMT, differentiation, and self-renewal in OECM-1 and SAS cells under normoxia and hypoxia.

A recent study revealed that IL-6 causes EMT in carcinoma cells 61. EMT is a key factor in the pathophysiology of fibrosis, and Collagen I is a key pathogenic factor in fibrosis 62. The pathological progression of EMT is decisively influenced by the presence of a persistent inflammatory microenvironment 42. TGF-β is a key modulator of fibrogenesis, regulates fibroblast phenotype and activity, induces myofibroblast transdifferentiation, and promotes matrix preservation 63. TGF-β is increased and activated in fibrotic disorders and is frequently associated with chronic inflammatory diseases, leading to compromised functions of vital organs. Our results confirmed that CoQ_0_ attenuated fibrosis (TGF-β and Collagen I) by inhibiting migration and EMT (increased E-cadherin and decreased Slug) in IL-6-treated SG cells.

The *in vivo* anticancer efficacy of CoQ_0_ (1.5 mg/kg) was confirmed using OECM-1 xenograft-bearing nude mice. In response to CoQ_0_, both tumor size and weight were dramatically decreased. In addition, body weight measurements and histological analysis of important organs were used in this investigation to determine the *in vivo* toxicity of CoQ_0_. No overt signs of harmful effects were found. The *in vivo* study data showed that CoQ_0_ therapy was efficient in preventing the onset of tumors and slowing tumor growth in OECM-1 xenograft-bearing mice.

This investigation found that noncytotoxic CoQ_0_ inhibited ROS (H_2_O_2_)-mediated HIF-1α expression in HNSCC (OECM-1 and SAS) cells. NLRP3 inflammation, metastasis (invasion or migration), and EMT were considerably reduced by CoQ_0_ treatment. Furthermore, CoQ_0_ decreased the Warburg effect by reducing aerobic glycolysis and enhancing mitochondrial oxidative phosphorylation in OECM-1 cells. Our study showed that the suppression of HIF-1α by CoQ_0_ played a pivotal role in the inhibition of the NLRP3 inflammasome, EMT/metastasis, and Warburg effect in HNSCC cells under both normoxia and hypoxia. CoQ_0_ attenuated fibrosis by inhibiting TGF-β and Collagen I production in IL-6-treated SG cells. Moreover, the anticancer efficacy of CoQ_0_ was confirmed* in vivo* due to the decreased tumor growth and size in OECM-1 xenograft-bearing athymic nude mice. The current investigation highlighted the therapeutic significance of CoQ_0_ in HNSCC cells and warrants further preclinical and clinical investigations to ascertain the benefits of CoQ_0_ treatment for head and neck squamous cell carcinoma patients.
